# A Guide to Nonaqueous Electrochemistry of f‑Element Complexes

**DOI:** 10.1021/acs.inorgchem.5c05041

**Published:** 2026-02-09

**Authors:** Julie E. Niklas, Matilda I. Duffy, Henry S. La Pierre

**Affiliations:** † School of Chemistry and Biochemistry, 1372 Georgia Institute of Technology, Atlanta, Georgia 30332-0400, United States; ‡ Nuclear and Radiological Engineering and Medical Physics Program, School of Mechanical Engineering, 1372 Georgia Institute of Technology, Atlanta, Georgia 30332-0400, United States; # Physical Sciences Division, Pacific Northwest National Laboratory, Richland, Washington 99352, United States

## Abstract

Electrochemistry is a powerful tool for assessing and understanding the redox chemistry of molecular complexes. Cyclic voltammetry enables the f-element community to study molecules in unusually high or low oxidation states, which potentially pose important broad-scope questions of electronic structure. In the pursuit of boundary-pushing compounds, reactive air- and moisture-sensitive species are often encountered, which can be challenging to characterize, especially when they are chemically incompatible with certain solvents, electrolytes, or electrodes or when their potentials lie outside of common electrochemical windows. Nonaqueous solvents and pseudo reference electrodes complicate many of the standard practices in acquiring high-quality and reproducible electrochemical data. This guide presents a detailed discussion of selecting appropriate cell conditions and referencing and addresses metrics for evaluating electrochemical and chemical reversibility. These methodological approaches have been extended to best practices for the electrochemical analysis of radioactive transuranic complexes.

## Graphical abstract

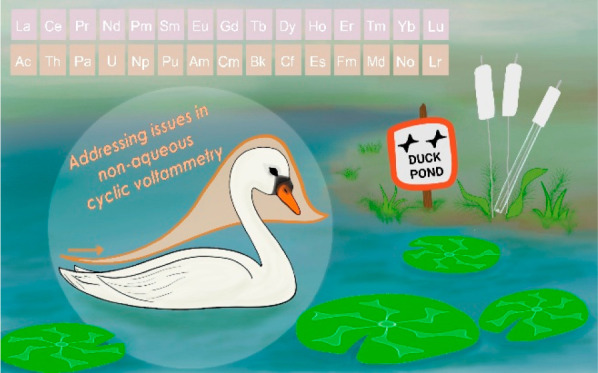

## Introduction

The electrochemical analysis of nonaqueous f-element systems is a key analytical tool to develop and study novel redox active f-element complexes. This analysis has facilitated the discovery and study of f-element ions in unusual oxidation states and coordination geometries.^1–16^ Nonaqueous molecular systems of both the 4f and 5f elements are areas of rapid development, with much attention devoted to accessing and understanding ligand effects on redox properties for both technical applications and fundamental chemistry. The development of ligands which can shift metal redox potentials into either the cathodic or anodic edges of solvent-accessible windows has enabled this progress in nonaqueous lanthanide and actinide chemistry. These shifts can result in redox potentials that lie just past the cusp of, or even far outside the chemical and electrochemical windows that are typically utilized or compatible with air- and moisture-sensitive complexes.^3,17^ These molecules are often nontrivial to synthesize and isolate and can be incompatible with some characterization methods under typical conditions (i.e., incompatibilities with solvents such as acetonitrile or dichloromethane, or with fluorinated electrolytes are not uncommon, especially under applied potentials).^18^ In this Viewpoint, we refer to such molecules simply as “reactive” for concision. Cyclic voltammetry is perhaps one of the most pertinent techniques for the study of such systems but can be fraught with logistical difficulties when probing analytes that are themselves potent reductants or oxidants.

The study of lanthanide complexes with metal ions in unusually low (2+, 1+, 0) and high (4+, 5+) oxidation states is an area of growing research interest, due to their unique chemical reactivity and physical properties, in contrast to the properties of the more ubiquitous lanthanide 3+ complexes.^1,6–9,11,19–28^ The study of actinides in aqueous acidic and basic media has been critical for applications in high-level waste streams and the nuclear fuel cycle.^29–38^ However, understanding some of these ions’ fundamental chemical and physical properties is dependent on the study of nonaqueous actinide (and lanthanide) complexes, and electrochemical analysis can define the redox properties of these systems.^17,39–44^ For example, the isolation and study of very low and high oxidation state of mid-actinide (U, Np, and Pu) complexes continues to reveal the intricacies of metal–ligand bond covalency, valence electronic structure, and redox chemistry.^2–5,13,45–54^ For transuranic species, due to their radioactivity and limited quantities available for elements past plutonium, there are additional constraints in handling, containment, and available amounts of analyte. As a result, electrochemical analysis can be more challenging. Thus, this guide is written in the context of f-element chemistry, since understanding properties of transuranic species can depend heavily on having robust, comparable data of analogous U or lanthanide complexes (i.e., why is the reversibility of a Pu complex so different from its Ce congener? ^3,4^ ).

The isolation and detailed characterization of low and high oxidation state f-element complexes rely not only on careful synthetic strategies but also on understanding their redox properties in nonaqueous solutions and in O_2_-free environments. As ligand design pushes redox potentials both cathodically and anodically, the field needs to not only identify wider electrochemical windows, but develop a common understanding of some of the variables that impact our ability to collect clean, standardized electrochemical data. The field currently lacks a concise experimental guide for selecting electrochemical cell conditions and setups, as well as for the acquisition and reporting of electrochemical data, especially when pseudoreference electrodes are employed. We note that, while this Viewpoint focuses on f-element complexes, many of the methodologies and techniques described here are applicable outside of the f-block.

Recently, the ACS has recognized the powerful role of electrochemistry in defining new complexes and published guidelines for the inclusion and presentation of electrochemical data, highlighting the widespread need for guidance in this area.^55^ We also direct the reader to Dempsey’s “A Practical Beginner’s Guide to Cyclic Voltammetry”,^56^ which covers the basics of topics such as electron transfer, Nernstian behavior, electrochemical cell components, referencing conventions, and reversibility, and assume the reader is familiar with these concepts. We also recommend Blakemore’s book chapter “Electrochemistry in Organometallic Chemistry”^57^ for an overview of electroanalytical methods and interpretation. This guide is intended to serve as a starting point for those exploring the nonaqueous electrochemistry of f-element complexes, particularly those requiring modified cell conditions, and to make accessible key data such as solvent and electrolyte windows, reference values of internal standards, and to demystify choices in referencing, electrodes, and cell setups for reactive and/or radioactive molecules. This guide is specific to electrochemical experiments conducted at room temperature in an inert-atmosphere glovebox with rigorous exclusion of air and water. We note that nonaqueous electrochemistry may be performed outside of a glovebox; however, the impacts of air and water can be important in experimental design and electrode preparations, and those aspects are not covered by this guide. Another topic not covered by this guide that may be of interest to readers is variable temperature (VT) cyclic voltammetry, especially for molecules that are unstable at room temperature or decompose faster than the timescale of many electrochemical experiments. VT electrochemistry can provide valuable quantitative thermodynamic data, although both cell kinetics and potentials are impacted by temperature.^58,59^

Herein, we report electrochemical windows for numerous organic solvent and electrolyte combinations and the potentials of four common metallocene reference compounds. These data were collected specifically for this Viewpoint, using the air-free methods reported here. Across the various solvent–electrolyte combinations, strong consistency was observed for each metallocene reference potential. We also include a brief list of best practices and additional notes on data collection in the Supporting Information (SI). This was developed as a practical, experience-based guide for glovebox-based electrochemistry of compounds that require small volume cells, particularly lanthanide and actinide complexes.

## Results and Discussion

### Solvent and Electrolyte Windows

Selection of appropriate solvent and electrolyte combinations can be difficult where strongly reducing or oxidizing compounds or oxo-/fluorophilic compounds are concerned. Here, we present a variety of solvent/electrolyte windows to aid in system selection. The utilized cell setup consists of a glassy carbon (GC) working electrode (WE), bare, fritted Ag^0^ wire in electrolyte solution as a (pseudo-) reference electrode (RE), and a Pt wire counter electrode (CE). We also direct the reader to Geiger’s discussions on the use of electrolytes with weakly coordinating anions and their impacts on thermodynamic and kinetic properties of redox reactions.^18,60–62^ Figure 1 plots the experimentally determined electrochemical windows for a range of solvent/electrolyte combinations. (See the SI for methods and data.) These values are tabulated along with a literature range^12^ in Table 1. We note that the potential of the ferrocene Fc^+/0^ couple ranges from +0.76 V to +1.26 V vs Ag^+/0^ in these systems (Table S1). Tetrahydrofuran (THF), while incredibly useful for many compounds, has one of the smaller windows when paired with tetra-*n*-butylammonium tetraphenylborate ([Bu_4_N]­[BPh_4_], TBABPh_4_). This smaller window can, at times, preclude the observation of both the cathodic and anodic waves of ferrocene, as this couple lies right on the edge of the window. Observation of the full ferrocene couple appears to be a balance among electrode positions, RE condition, ferrocene concentration, and *iR* (internal resistance) compensation. More information on *iR* compensation can be found in Dempsey’s guide.^56^ Using the standard setup described herein, we find that lower ferrocene concentrations perform better, and the use of appropriate *iR* compensation values (see the SI) is advantageous for observing the Ep_a_, anodic peak potential. This limitation in THF can be overcome through the use of tetra-*n*-butylammonium hexafluorophosphate ([Bu_4_N]­[PF_6_], TBAPF_6_), which expands the window.

**Figure 1:**
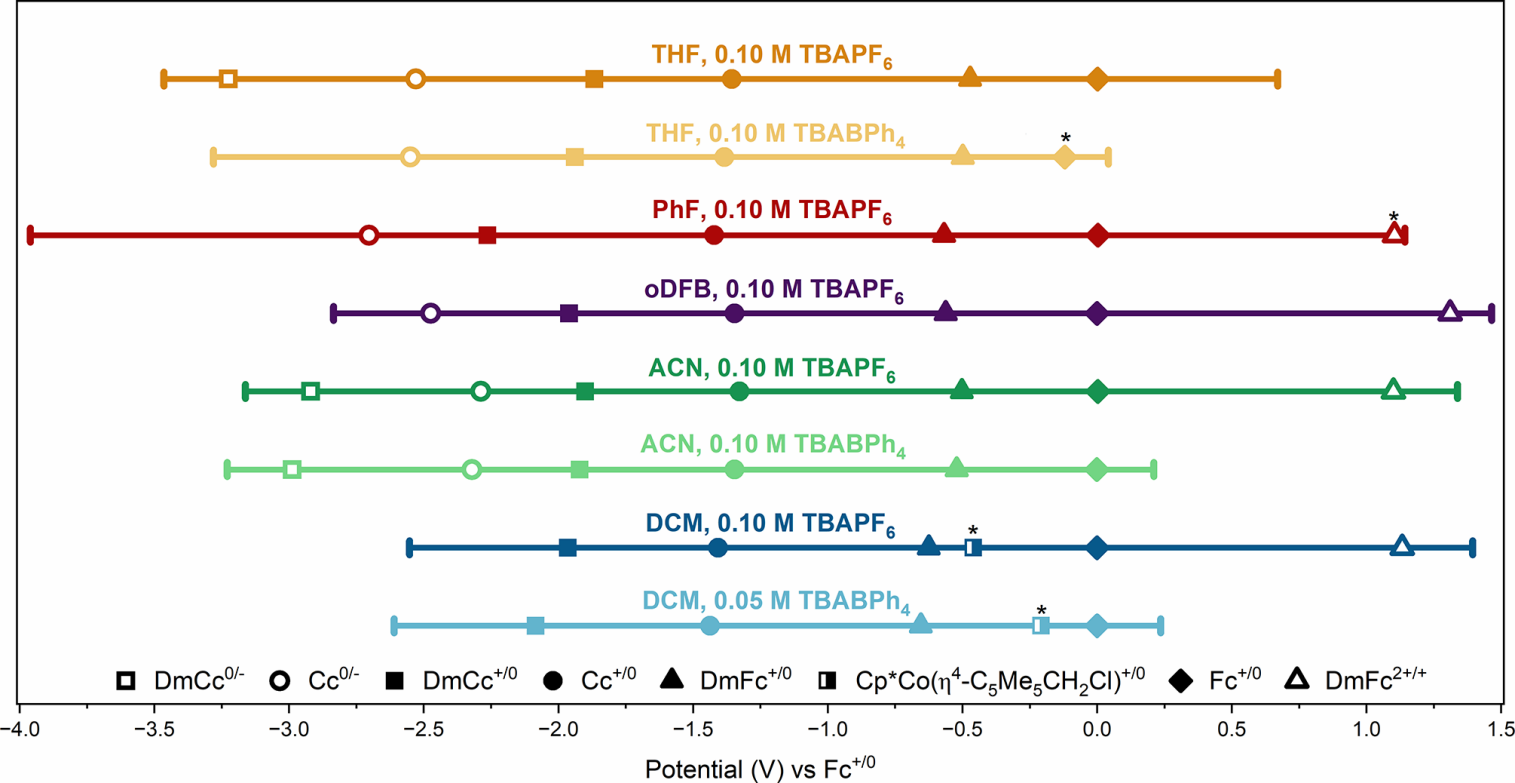
Established windows and *E*_1/2_ values of metallocene reference compounds for various solvent/electrolyte combinations. WE: GC, 3 mm; RE: bare Ag^0^ wire, fritted, in electrolyte solution; CE: Pt wire, 0.5 mm. Scan rate: 200 mV/s. Metallocene concentrations are approximately 0.1 to 0.2 M. *Denotes Ep_c_ values for Fc^+/0^ and DmFc^2+/+^ and Ep_a_ values for Cp*Co­(η^4^-C_5_Me_5_CH_2_Cl)^+/0^.^73^

**Table 1:** Potential Ranges (V) of Solvent/Electrolyte Combinations

	Approx. Electrochemical Windows (V vs Fc^+/0^)	
	Cathodic limit	Anodic limit
**THF/TBAPF**_**6**_	–3.5	+0.7
**THF/TBABPh**_**4**_	–3.3	0.0
**THF/TPABArF**_**24**_ ^a^	–2.3	+1.9
**PhF/TBAPF**_**6**_	–4.0	+1.1
**oDFB/TBAPF**_**6**_	–2.8	+1.5
**ACN/TBAPF**_**6**_	–3.1	+1.3
**ACN/TBABPh**_**4**_	–3.2	+0.2
**DCM/TBAPF**_**6**_	–2.6	+1.4
**DCM/TBABPh**_**4**_	–2.6	+0.2
**DME/TBABF**_**4**_ ^b^	–3.5	+1.7

a From ref 12, measured with Au WE, fritted Ag/AgCl RE, Pt wire CE.; this range is from blank electrolyte. All others are from solutions containing four metallocene standards. All electrolyte concentrations are 0.10 M, with the exception of DCM/TBABPh_4_ (0.05 M).

b Values are approximations based on SI plots—the window may extend past these values.

Fluorobenzene (PhF) has one of the largest and most negative examined electrochemical windows, but TBABPh_4_ is not soluble and, therefore, is not a viable electrolyte. Similarly, *ortho*-difluorobenzene (oDFB) does not solubilize TBABPh_4_, but it does have a moderately increased positive range over PhF. For these solvents, other electrolytes featuring BArF_*n*_^–^ (ArF_*n*_ = C_6_F_5_, C_6_H_3_(3,5-CF_3_); *n* = 20 or 24, respectively) or tetrafluoroborate (BF_4_^–^) anions may be advantageously soluble.^18^ TBABArF_24_ has also been shown to enhance the reversibility of certain metallocenes in dichloromethane (DCM).^60^ BArF_*n*_^–^ salts are excellent electrolytes which have generally wide windows and good compatibility with analytes—their downside is cost, which may be prohibitive to some; however, their preparation has been documented.^63^ We suggest that it may be advantageous for the community using these salts to document their windows in publications. Additionally, electrolytes containing tetrapropylammonium (^*n*^Pr_4_N^+^) or tetraethylammonium (Et_4_N^+^) cations may provide larger electrochemical windows than tetrabutylammonium salts.^63–66^ The electrolyte [^*n*^Pr_4_N]­[BArF_24_] possesses a fairly large window, and reaches some of the most positive potentials as previously reported.^12,17^ In dimethoxyethane (DME), TBABF_4_ is shown to have a rather large electrochemical window, covering at least the range from approximately −3.5 V to +1.75 V vs Fc^+/0^.^58^ As seen in all cases, TBAPF_6_ electrolyte boasts larger windows than TBABPh_4_, which can be oxidized at sufficiently anodic potentials;^67^ however, not all compounds may be compatible with the PF_6_^–^ anion. Fluoride abstraction (accessible from the [PF_6_]^−^ ⇌ PF_5_ + F^–^ equilibrium) can lead to reactions with low-coordinate fluorophilic metals or electron-poor ligands.^18,62^ Acetonitrile (ACN), while providing a fairly large electrochemical window when paired with TBAPF_6_, is not compatible with many reactive analytes.

Where possible, we recommend using electrolyte concentrations that are 100 to 200 times greater than the analyte concentration (approximately 0.10 or 0.20 M) to reduce the *iR* of the solution and minimize *iR* drop (ohmic drop) during the measurement. Exceeding the upper limit of the electrolyte concentration can lead to unwanted effects on ion mobility and increased solution viscosity. In the case of DCM, TBABPh_4_ is only soluble to just over 0.05 M, and in THF, it is soluble up to 0.10 M. The dissolution in THF is kinetically slow and requires time and significant agitation to dissolve completely. For THF, in most cases, higher electrolyte concentrations help counteract the low conductivity of the solvent and should be used when possible. Increased electrolyte concentrations in some solvents may allow for slight expansions of the electrochemical windows, as well. As an additional note, the best practice is to prepare each solution immediately before the measurement. Storage of stock solutions is convenient but can lead to electrolyte degradation. For example, TBABPh_4_ in THF degrades overnight, and clean scans of the electrolyte solution are not achievable. We also note that this electrolyte is costly but can be recovered and used again, although additional recrystallization steps may be necessary for purification. (See the SI.) Therefore, we encourage the collection of full, blank electrochemical windows to evaluate the cleanliness of the electrolyte solution prior to data collection. We also note that the inclusion of these scans in the SI can be helpful in establishing the electrochemical window under the conditions applied, should authors choose to include them.

### Metallocene Standard Potentials

It is important to include measurement of an internal and/or external standard to gauge electrode drift and verify the RE, particularly when utilizing a pseudoreference or fritted electrode. An ideal standard should have a potential that does not overlap with that of the analyte and should be inert to reactivity with the analyte. Table 2 compares the experimentally determined potentials (vs Fc^+/0^) for four common metallocene standards (ferrocene ([Fe­(Cp)_2_], Fc), decamethylferrocene ([Fe­(Cp*)_2_], DmFc), cobaltocene ([Co­(Cp)_2_], *Cc*), and decamethylcobaltocene ([Co­(Cp*)_2_], DmCc)) across a range of solvents and electrolytes. These data can be readily assigned and are consistent with previously reported values.^68–70^ Reported data were collected with all four standards in solution at once, with the exception of PhF/TBAPF_6_, for which data were first collected without DmCc then a second window was collected upon the addition of DmCc. It is worth noting that all of the measured metallocene standards have quasireversible couples under the utilized conditions. This is due to larger uncompensated internal resistance in nonaqueous solvents, and the use of a pseudoreference electrode.

**Table 2:** Potentials (V) of Metallocene Standards in Each Solvent/Electrolyte Combination Referenced to Fc^+^/Fc at a Scan Rate of 200 mV/s

	DmCc (0/−)	*Cc*(0/-)	DmCc (+/0)	Cc(+/0)	DmFc (+/0)	Fc (+/0)	DmFc (2+/1+)
**THF/TBAPF**_**6**_	–3.23	–2.53	–1.87	–1.36	–0.47	0.00	–
**THF/TBABPh**_**4**_	–	–2.55	–1.94	–1.38	–0.50	–0.12^a^	–
**PhF/TBAPF**_**6**_	–	–2.70	–2.26	–1.42	–0.57	0.00	1.10^a^
**oDFB/TBAPF**_**6**_	–	–2.47	–1.96	–1.35	–0.56	0.00	1.31
**ACN/TBAPF**_**6**_	–2.92	–2.29	–1.90	–1.33	–0.50	0.00	1.10
**ACN/TBABPh**_**4**_	–2.99	–2.32	–1.92	–1.35	–0.52	0.00	–
**DCM/TBAPF**_**6**_	–	–	–1.97	–1.41	–0.63	0.00	1.13
**DCM/TBABPh**_**4**_	–	–	–2.08	–1.44	–0.65	0.00	–

a Denotes and Ep_c_ value. All electrolyte concentrations are 0.10 M, with the exception of DCM/TBABPh_4_ (0.05 M). Values reported are for solutions containing internal standards.

Ferrocene is arguably the most common standard and used in this work as the reference for the rest of the standards. The difference in potential between the Fc^+/0^ and DmFc^+/0^ couples is well established to be approximately 0.5 V,^3,4,69^ which our data corroborates across a variety of solvents and two different electrolytes. When the potential of an analyte’s redox couple falls between −0.5 and 0.0 V, vs Fc^+/0^, Fc and DmFc may not be appropriate internal standards. Cc and DmCc have more negative potentials and can be used in these cases.

In solvent/electrolyte conditions containing all four standards, oDFB/TBAPF_6_, ACN/TBAPF_6_, and DCM/TBAPF_6_ have an additional feature at 1.31 and 1.10 V, and 1.13 V, respectively, facilitated by the larger electrochemical windows (see Figure 1, as well as Figures S13, S16, and S19). This couple is quasireversible and not well-defined, but can be assigned as the DmFc^2+/1+^ (Fe^4+/3+^) couple, based on limited literature precedent.^71–73^ The Ep_c_ of this feature is also visible in PhF/TBAPF_6_ at 1.10 V (Figure S11). The DmFc^2+/1+^ couple is often inaccessible in organic solvents due to the highly anodic potentials and limitations of electrochemical windows. Gonzalvez et al. utilized ionic liquids to observe the DmFc^2+/1+^ potential at 1.8 V vs Fc^+/0^ using glassy carbon and boron-doped diamond electrodes.^71^ Similarly, liquid SO_2_ at −40 °C was employed by Sharp and Bard revealing this couple at approximately +1.3 V vs Fc^+/0^, using a Pt disc WE.^72^ Notably, the DmFc^1+/0^ couple is reported at approximately −0.2 V vs Fc^+/0^ under these conditions. Gale and Singh^73^ also report the DmFc^2+/1+^ potential at approximately +1.4 V vs Fc^+/0^ in a 1.5/1 aluminum chloride/1-butylpyridinium chloride melt using vitreous carbon and aluminum wire electrodes. Mills et al. observe an irreversible DmFc^2+/1+^ couple at 1.06 V vs Fc^+/0^ at −50 °C in DME using [^*n*^Bu_4_N]­[BF_4_] electrolyte, platinum wire WE, and AgCl/Ag wire pseudo-reference electrode.^58^

DmCc offers the most negative reference potentials, ranging from −1.97 V to −1.87 V vs Fc^+/0^, with the exception of DmCc in PhF/TBAPF_6_ and DCM/TBABPh_4_, in which the potential falls at −2.26 V and −2.08 V, respectively. The addition of DmCc to the PhF/TBAPF_6_ solution results in a significant increase in uncompensated iR, which yields a voltammogram with greater Δ*E*_p_ values for all standards (Figure S11). Regardless of the Δ*E*_p_, the center of the couple for each standard remains the same in the presence and absence of DmCc, with the exception of the Cc^–/0^ couple (Table S2). The DmCc^0/–^ couple is not observed, despite the fact that PhF/TBAPF_6_ has the most negative window. This effect is due to the broadening of all features induced by DmCc; the Cc^0/–^ feature most likely overlaps with the DmCc^0/–^ feature, as corroborated by the change in *E*_1/2_ for Cc^–/0^. Importantly, DmCc has reported reactivity with DCM,^74^ which may influence the effectiveness of this standard when collecting data in this solvent. Blakemore reports Cp*Co­(η^4^-C_5_Me_5_CH_2_Cl) as the product of the DmCc and DCM reaction, which has an irreversible feature at an Ep_c_ of approximately −0.5 V vs Fc^+/0^.^74^ In DCM/TBAPF_6_ and DCM/TBABPh_4_, we report Ep_c_ values at −0.46 V and −0.21 V vs Fc^+/0^, respectively (see Figures S20, S22, and S23). In DCM/TBAPF_6_, the Cp*Co­(η^4^-C_5_Me_5_CH_2_Cl)^+/0^ feature overlaps with the DmFc^+/0^ feature, and the reported DmFc^+/0^ *E*_1/2_ (Table 2) was taken from a scan in the absence of DmCc (Figure S20).

### Electrodes and Cell Setup

The following considerations are outlined and have been optimized for the study of reactive complexes (including some transuranic ions) in small volumes. A standard cell setup for work in an inert atmosphere glovebox consists of a 20 mL glass scintillation vial containing 5 mL of electrolyte or analyte solution fitted with a Teflon cap through which the WE, RE, and CE are inserted (Figure 2). The cap may be secured to the vial with parafilm for stability. With this Viewpoint, we provide CAD (computer-aided design) files for Teflon vial caps which fit standard working and reference electrodes, and straight Pt wire electrodes. These caps allow standard 20 mL scintillation vials to be used as the cell and have the added benefits of being disposable (if needed) and are easy to machine from a Teflon rod. The use of these caps also allows the user to fix the electrodes at an appropriate height within the solution, and serves to immobilize the electrodes, which avoids disruption of the double layer and linear diffusion by convection. The electrode positions are optimized to place the RE inline between the WE and CE to minimize *iR* drop.

**Figure 2:**
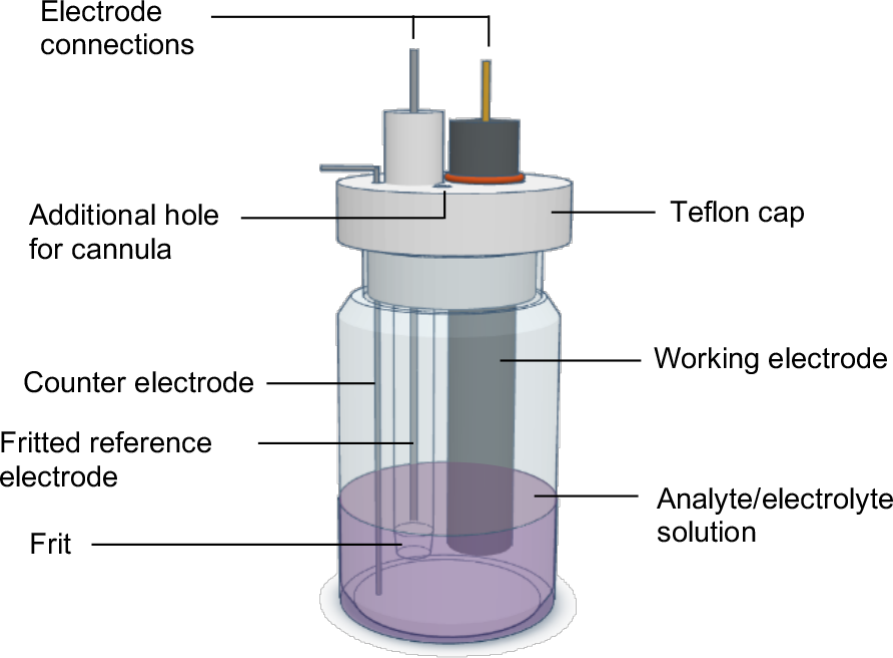
Depiction of a three-electrode electrochemical cell optimized for a 20 mL scintillation vial and 5 mL of solution. Shown is a sheathed glassy carbon WE, a fritted Ag wire RE and straight Pt wire CE. An additional hole in the cap for cannula insertion allows for gas or reagent addition.

GC electrodes are one of the most popular choices for a WE in nonaqueous electrochemistry and are generally compatible with a wide range of analytes. Though these are the focus of this Viewpoint, we note that other WEs, such as Au and Pt discs, may be employed successfully and will likely change the electrochemical windows and may change observed analyte potentials. An ideal GC WE should be a shiny, dark, and reflective surface with no visible scuffs or pitting. Between analytes, or between experiments in the case of electrodeposition during the course of analysis, the electrode should be cleaned gently with the experimental solvent, then polished on a microvelvet pad mounted to a flat surface with a fine alumina slurry, using rotating figure-8 strokes, and cleaned again gently with solvent. Air- and moisture-free alumina slurries can be prepared by suspending dried alumina, 0.05 μm, (see the SI) in a nonpolar organic solvent such as hexanes. When necessary, the electrode can be removed from the glovebox and sonicated briefly, although we note that extensive sonication can, over time, lead to severe pitting and degradation of the GC surface, so should be done in moderation or in a solvent such as 1,4-dioxane.^75,76^ Despite the range of processes available to repair a GC WE electrode, at some point, it should be retired, for example, when sonication, repolishing, and even sanding/refinishing then repolishing fail to improve electrochemical response.

Many GC WEs are now produced at a more affordable price point with PTFE (polytetrafluoroethylene) sheaths. PTFE is generally compatible with many compounds, but extremely reducing compounds may require the use of a PCTFE (polychlorotrifluoroethylene) sheath. Particularly in conjunction with bare Ag wire pseudoreference electrodes, plating and destruction of PTFE sheaths has been observed when scanning to negative potentials. As a general guideline—if a compound reduces Teflon liners (vial caps, tube liners, spatulas, and Teflon-coated stir bars), it is best to avoid a PTFE WE sheath.

Pt wire CEs are simple to clean but should be handled with care to avoid breaks and kinks. In the glovebox, wiping firmly but gently with a Kimwipe and solvent is sufficient to clean between analytes. Pt wire can be cleaned outside the glovebox by soaking it in concentrated HNO_3_ for a few minutes and then rinsing with water and acetone. Since there is a range of CE body types one may encounter, including sheathed or unsheathed, coiled, straight, and meshes, be sure to consider the physical space needs within the electrochemical cell and cap, and the ease of cleaning. In this regard, straight, bare wires are the easiest to clean (and decontaminate in the case of radioactive analytes), but other types typically provide more CE surface area. As a general rule of thumb, the surface area of the CE should be at least 10 times greater than that of the WE as to not rate-limit processes at the WE and to reduce *iR* drop, which is why many Pt CEs are coiled.^77^ A straight Pt wire (0.5 mm diameter) submerged entirely in 5 mL of solvent (approximately 13 mm) inside a 20 mL scintillation vial of solution provides only about 3× the area of the WE (3 mm diameter); however, this is a tradeoff when other considerations are prioritized. Smaller solution volumes require a smaller vessel to increase the submerged surface area of the CE.

There are many methods to prepare Ag wire reference electrodes, depending on if a true reference electrode or a pseudoreference electrode is desired. This guide focuses on the latter, as pseudo-referencing is the predominant technique in inert atmosphere, nonaqueous systems, where an internal standard such as ferrocene is used to calibrate the measured redox potentials. Pseudoreference Ag electrodes often take the form of either a coated Ag/AgCl wire or a bare Ag wire. Additionally, the Ag wires can be used with or without a frit, discussed below; however, submerging bare wires in an analyte solution under an applied potential is not recommended, as deleterious interactions may result. The Ag/AgCl electrode is prepared by polishing silver wire with fine grit sandpaper such as SiC to remove any oxide layer, and submerging it in concentrated HCl (or similar Cl^–^ source, such as FeCl_3_) for several minutes. Reforming this AgCl layer on the Ag/AgCl electrode frequently (every few analytes) is best; however, the coating should be quite robust. The wire can then be rinsed with water and other solvents, patted dry, and taken into the glovebox. Alternatively, a bare Ag wire can be prepared by polishing/sanding inside the glovebox to ensure no oxide layer forms, wiping it clean, and using it directly in the cell or with a frit. When used without frits, Ag^0^ or AgCl wires should be cleaned between analytes with a Kimwipe and solvent. Both types of Ag wires can be stored dry. It is important to note that the Ag/AgCl wire will be more stable toward electrode drift and uncompensated resistance than a bare Ag^0^ wire. Still, excellent results can be obtained with both, and in the cases of radioactive analytes, the Ag^0^ wire may be a better choice as the electrode does not need to be removed from the box for re-treatment.

When Ag reference electrode frits are used, it is important to know what type of frit you are using. There are two prevalent styles of frits: (1) a glass tube with one open end and an inset ceramic frit in the other and (2) a glass tube open on both ends to which a Vycor (discontinued) or Varapor porous glass frit is affixed with FEP (fluorinated ethylene propylene) heat-shrink tubing. Either can be dried in an oven overnight before initial use, but it is possible that Vycor or Varapor may interact with some analytes and should be tested for compatibility before use. Once a frit has been oven-dried and used, do not let it dry again—it should be submerged continually in one electrolyte and one solvent at one concentration. If additional solvents, electrolytes, or concentrations are needed, additional frits for each condition must be prepared.

While a classic Ag/AgCl or bare Ag^0^ wire pseudoreference electrode may be successful for some compounds, interactions between the analyte and the electrode cannot be ruled out, especially for reactive complexes. This electrode reaction has been observed in f-element imidophosphorane complexes, where changes in *E*_1/2_ values of over 500 mV have been recorded across reference electrode types (see Figures S24 and S25). A frit filled with electrolyte solution around a bare silver wire is a great option in these cases, as the frit limits mass transport and therefore interactions between the Ag surface and the analyte. The introduction of a frit does create a liquid junction potential ((LJP), or a diffusion potential drop when solvent is the same) and can increase the likelihood and/or degree of reference electrode drift. Drift may need to be accounted for in the data workup. On the other hand, with some analytes, using an internal standard such as ferrocene in the presence of a bare Ag/AgCl wire leads to unreliable referencing, and can shift the Fc^+/0^ couple as much as 1 V, effectively offsetting the observed analyte redox couple by as much (Figures S26 and S27) or it can prevent the observation of the Fc^+/0^ couple altogether (more on referencing below). These observed shifts in analyte *E*_1/2_ and ferrocene reference values demonstrate how critical aspects of cell setup can be—these aberrant features can invalidate the referencing and left unchecked could quickly result in erroneous data interpretation. We suggest the use of internal references that do not overlap with analyte redox events, and, in the case that an interaction between analyte and reference is suspected and alternative method of referencing is employed. The proper maintenance of electrodes is critical to acquiring high-quality electrochemical data. Generally, electrodes can be both stored and cleaned in the glovebox and should always be cleaned between analytes.

### Internal and External Referencing

Referencing electrochemical data for reactive nonaqueous systems, especially when a pseudo-reference electrode is used, can be challenging. Pseudo-reference electrodes do not generally have stable or reproducible redox potentials, so internal standards such as ferrocene, decamethyl ferrocene, cobaltocene, or decamethylcobaltocene are employed. When studying reactive molecules or those with potentials at the boundaries of the electrochemical window, interactions between the analyte and internal standard are a concern, especially if an unfritted RE is used (see Figures S26 and S27). For fritted REs, this may be less of an issue, but caution should still be exercised when adding an internal standard to the analyte. As such, we suggest the use of both an external standard as well as an internal standard after all necessary data have been collected. This process requires preparing two vials at the beginning of the experiment (Scheme 1)—one of which contains just electrolyte and one which contains electrolyte and the analyte. We recommend adding solvent to these vials only immediately before each is measured, as there may be some reactive analytes that degrade on the timescale of the experiments in the chosen solvent. The electrodes and cap are transferred between the two vials for the respective measurements. The electrodes should be rinsed with solvent and gently wiped while being moved between vials.

**Scheme 1:**
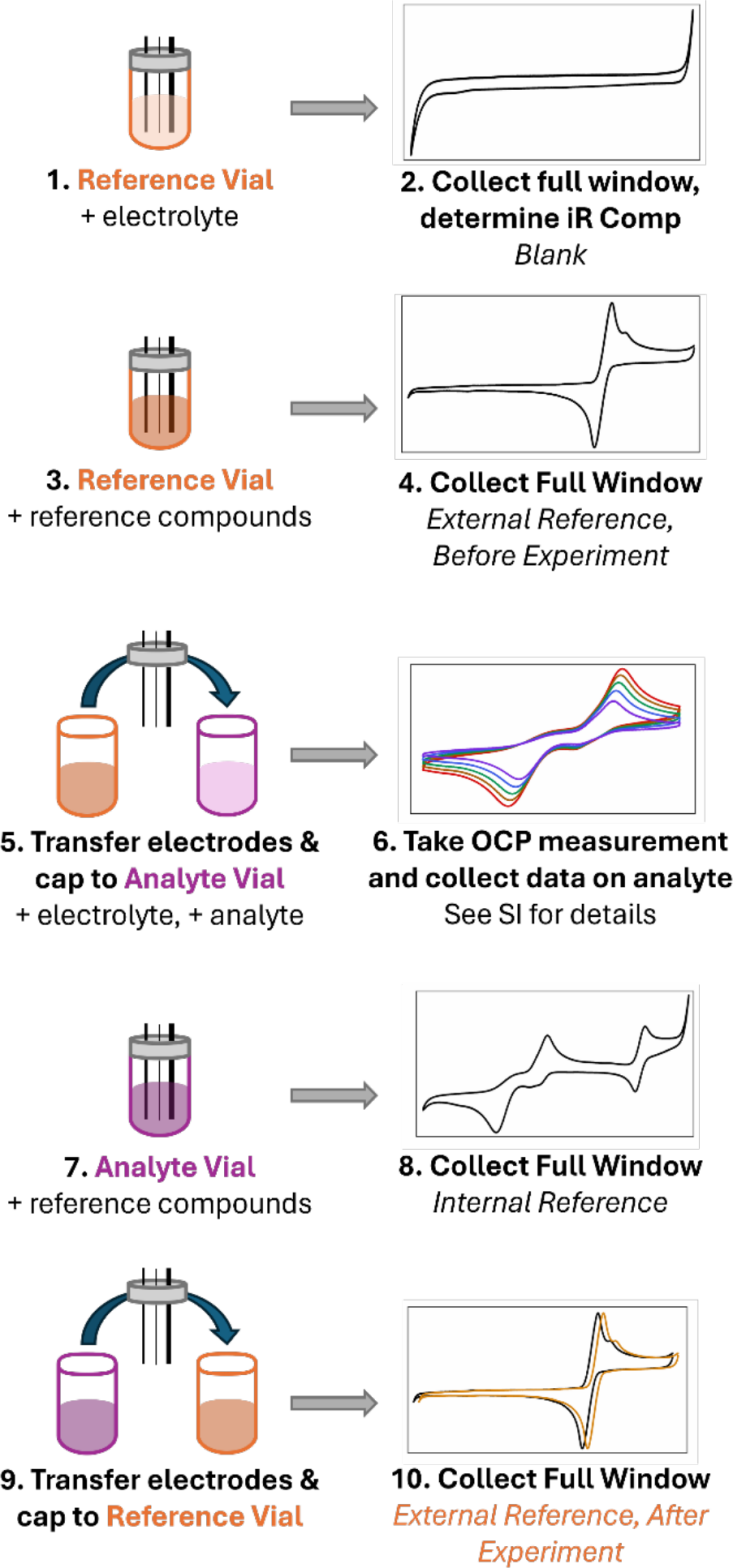
Workflow for Using a Two-Vial System to Collect and Reference Data^a^

a Note: this does not capture the full range of experiments that one may need to perform on their analyte.

The electrolyte-only vial is used to establish a blank or background scan to document cleanliness of the electrolyte and establish the electrochemical window and any needed *iR* compensation for the experiment. A small amount of internal standard(s) can then be added, and redox potentials can then be measured externally, both before and after the experiment. Comparison of the “before” and “after” measurements is useful to assess if and/or how much electrode drift has occurred during the experiment and if manual correction is required. In cases where the internal standard interacts poorly with the analyte,^78^ an average of the two external standard measurements can often be used. Over a series of imidophosphorane complexes in which ferrocene is a compatible standard, we have observed drifts ranging from 0 to 120 mV over the course of the experiment, for which the average of the external standard *E*_1/2_ values differ from the internal standard by 20 mV or less (see Tables S3 and S4). Considerably larger differences were observed when using an unfritted Ag/AgCl electrode (230–980 mV). A fritted Ag/AgCl RE should be comparable or superior to a fritted bare Ag^0^ wire in this regard. When possible, values referenced to an internal standard should be reported and the referencing method should be clearly documented.

### Assessing Reversibility in Non-Ideal Systems

Discussing the electrochemical reversibility of couples in nonaqueous solvents, and with the use of pseudo-reference electrodes/small-surface area CEs, poses a challenge, as the Ep_a_/Ep_c_ separations tend to be large, even when using *iR* compensation. Even internal standard metallocenes for which electron transfer is typically highly reversible (fast), the ΔEp values can span from around 63 mV (at their smallest, DmFc^+/0^) to roughly 800 mV (at their largest) (Table S2). Most of these fall well outside of the ideal 57 mV ΔEp which classically defines a chemically and electrochemically reversible system (Nernstian), but this is not typically discussed when assigning the reversibility of redox couples under less standard conditions—in many cases, electron transfer processes are simply dubbed “quasireversible” when they have a ΔEp larger than 57 mV. The language of reversibility fails us somewhat when analyte couples are as “reversible” or more “reversible” than those of reference compounds under non-ideal conditions.

In evaluating the reversibility of a couple, one must consider not just the rate of the electron transfer but the rate of electron transfer *relative* to charge and mass transport in the system. Standard analyses should not necessarily invoke Butler–Volmer kinetics or steady-state mass transport analysis, especially as nonaqueous systems can require challenging adaptations of these models. However, electrochemical reversibility cannot be evaluated strictly on an adherence to a 57 mV peak separation in many nonaqueous systems (however, there are systems in which ideal behavior can be observed). One indication of reversibility is an invariance of *E*_1/2_ values with scan rate. ΔEp, however, is determined by a combination of kinetic effects (Ep will always vary with scan rate) and uncompensated resistance in the electrochemical cell, and even with *iR* compensation, there is fundamentally always at least a few ohms of uncompensated resistance.^79^ In many cases, its impact will be negligible. These factors can mimic responses which are found in systems where electron-transfer kinetic limitations need to be considered (e.g., quasireversible systems).^79^ An additional consequence of these factors is that the line between a quasireversible process and multielectron process can become blurred, as the simplistic 57 mV/*n* rule does not always hold and may necessitate use of an internal standard or other electroanalytical methods.^80^ In practice, few, if any, of the conditions for true electrochemical reversibility will be met in most pseudo-referenced and/or nonaqueous systems. How, then, in standard analyses do we assign such redox couples as irreversible, quasireversible, or reversible and disentangle the effects of chemical reversibility (or lack thereof)?

Electrochemical irreversibility is perhaps the easiest case to identify—either there is no return feature or the return feature is displaced significantly, elongating or entirely disrupting the ideal “duck” shape. When the analyte is known to be stable in both oxidation states with minimal rearrangement between the two, a large ΔEp indicates slow electron transfer. Larger rearrangements may be indicated by additional features, asymmetry, and/or even larger ΔEp values. In some cases, where a process is irreversible due to a chemical step following electron transfer (chemically irreversible), the return couple may be absent or may only be evident at high scan rates where the timescale of the experiment outcompetes the rate of the subsequent chemical step.^81^ A plot of log_10_(ν) vs Ep (where ν is the scan rate) will be linear for irreversible processes. Deviations from linearity will warrant further investigations into the nature of the process.

Quasireversibility, as the name suggests, occupies an intermediate regime, where some aspects of reversibility are retained but not others. Chemical reversibility may persist, while electrochemical reversibility does not, depending on how asymmetric the peaks are and how great ΔEp is. Randles-Ševčík plots (*i*_p_ vs ν^1/2^) which deviate significantly from linearity or are largely asymmetric indicate quasireversibility. However, so do large ΔEp values, making many processes, which, at first glance, may seem reversible, best classified as quasireversible. Some authors will classify any couple with a ΔEp larger than 57–60 mV as quasireversible out of an abundance of caution. However, this approach potentially obscures the nature of the observed chemical process since, due to normal experimental deviations from ideality, couples with ΔEp values of even 70 mV can, in fact, be reversible.

Evaluating electrochemical reversibility poses a greater challenge—where these nonideal systems are concerned—simply determining the peak splitting and scan rate dependence is not a reliable method. In these cases, one is tempted to invoke the concept of *practical reversibility* as put forth by Bard, which “is not an absolute term; it includes certain attitudes and expectations an observer has toward the process.”^79^ One could posit that if some of these nonideal systems could reliably be measured, say, in acetonitrile, with a true reference electrode, they may in fact exhibit Nernstian behavior. When the experimental conditions are changed, classically reversible processes can become quasi- or irreversible by nature of the measurement. Maybe, then, it is appropriate that electron transfer events measured under nonaqueous, pseudo-reference conditions which behave *almost* reversibly, may in practice be called reversible, or perhaps near-reversible.

Clearly, variations in experimental conditions can cause large variations in ΔEp values—we have good ways to establish reversibility in more ideal systems, but when such large deviations from ideal conditions are employed, reversibility is an unrealistic expectation. To ascertain assigning reversibility in a practical sense the following should be considered:^82^

(1) Symmetry about the vertical axis of the couple is an indication of chemical and electrochemical reversibility. Likewise, a peak current ratio closer to one indicates a more chemically reversible system;^74^
(2) when products are chemically stable, but electron transfer is slow, the symmetry of the couple about the vertical axis may be lost, indicating quasireversibility;
(3) When the electron transfer product itself is not stable, return currents are often diminished—this is a further sign of irreversibility.^82^

More-detailed analysis is also useful and recommended. Randles-Ševčík plots (i_p_ vs ν^1/2^) should be generated when the assessment of reversibility is important. Linearity and symmetry are indicators of reversibility, however slowed electron-transfer and/or uncompensated resistance can perturb these. As such, we suggest in discussions of reversibility that authors define the scope and considerations in which they are making assignments, and put forth sufficient data with which to substantiate them. While practical reversibility is a useful qualification, it is still most prudent to be conservative in assignments, as the thermodynamics and kinetics of electron transfer in nonaqueous, pseudo-referenced systems will rarely meet the conditions for true electrochemical reversibility.

As a case study, DmFc, which has been touted as a superior internal standard for its reversibility and solvent independence,^78^ was measured in THF/TBABPh_4_ (0.10 M) with a GC WE, fritted Ag wire RE, and Pt wire CE (Figure 3A). Some may be tempted to designate this as a quasireversible couple, due to its ΔEp values (77 mV at 50 mV/s). From the scan rate dependence study, a Randles–Ševčík plot was generated (Figure 3B), showing symmetric, linear relationships between *i*p and ν^1/2^, both of which indicate reversibility. In a practical sense, this is a reversible, well-behaved redox couple with relatively fast electron transfer kinetics under the conditions, despite its slightly greater than the expected ΔEp. This system highlights the careful consideration necessary for the assignments of reversibility and quasireversibility.

**Figure 3:**
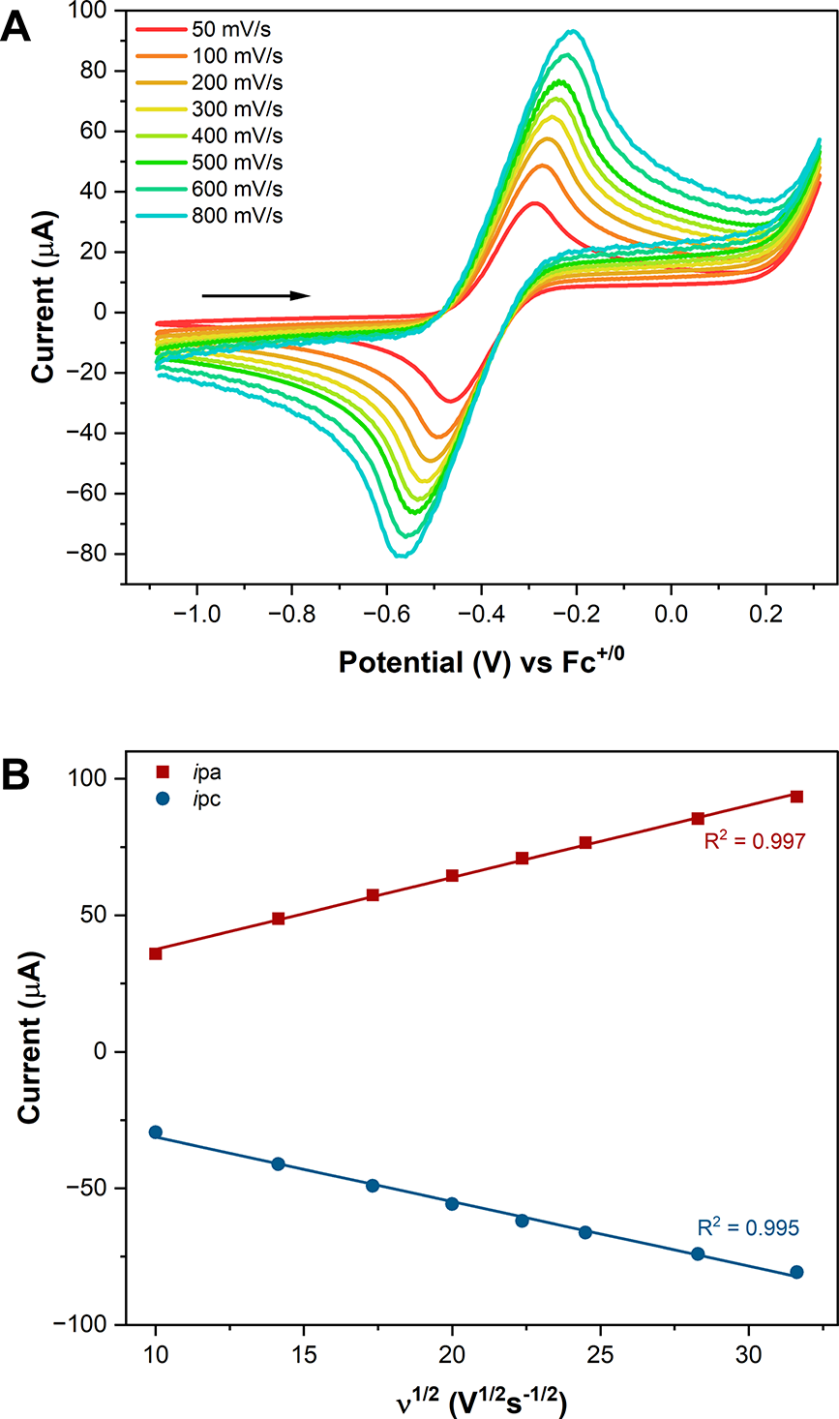
(A) Decamethylferrocene (DmFc) measured at varying scan rates in THF with 0.10 M TBABPh_4_ supporting electrolyte. WE: GC; CE: Pt wire; RE: fritted Ag^0^ wire. (B) Randles-Ševčík plot for peak currents of DmFc couple at varying scan rates.

### Considerations for An/TRU

The demands of transuranic (TRU) chemistry can impose restrictions on aspects, such as cell setup, reusability of components, and analyte concentrations. As the availability of isotope decreases, so does the amount of analyte, which can be dedicated to electrochemical experiments, where the eventual recovery of isotope from electrolyte poses additional complications. Maintaining useful analyte concentrations for use with macroelectrodes (as opposed to ultramicroelectrodes and smaller systems) therefore requires the use of low cell volumes: 5 mL in a standard 20 mL scintillation vial works well for U, Np, and Pu, provided certain electrode conditions are met, and for Am and later actinides, volumes of 1 mL or possibly lower are likely requisite. Meeting the requirements of a three-electrode electrochemical cell can be difficult, where small volumes are required. Additionally, detailed planning of electrochemical experiments is necessary, as repeat experiments are not often performed on transuranic species due to the cost and availability of these isotopes. If data are compared to a lanthanide or U/Th model systems, the same cell and electrode conditions should be employed when possible. Though not always practical, data on model systems can be useful in guiding how (and which) electrochemical experiments are performed on transuranic species, especially if simulations are to be used in data analysis.

Though this applies to a smaller community, it is one that relies on electrochemical information heavily, and has already established precedent in how we construct electrochemical experiments on transuranic analytes.^3,4,12,17,43,65,83,84^ There are a number of considerations which are important to transuranic electrochemistry.

(1) The electrochemical cell should be designed in such a way to minimize risk of contamination of the work area and should be physically stable and easy to manipulate and clean. The use of a cap through which the electrodes are inserted is especially beneficial here. CAD files for open-cap disc inserts for use with 5 mL conical vials are also provided in the SI. Additionally, the solution should have sufficient depth to maintain a CE surface area 10 times greater (ideally) than that of the WE, as well as sufficient headspace such that insertion of the electrodes does not displace the solution too near the top of the cell. Here we recommend a bare straight Pt wire CE for ease of cleaning and decontamination and a 3 mm WE. Smaller WEs which maintain a higher CE:WE area ratio are available but can be hard to find and costly. The use of a bare Pt wire allows all of the electrodes to fit into the diameter of a 20 mL scintillation vial or a 5 mL conical vial.
(2) The ideal cell and its components are disposable and should not be used across isotopes. For TRU analytes in particular, we do not recommend reusing an electrochemical cell because its cleaning and storage poses a contamination risk to the work area.
(3) Isotope quantities (and, thus, analyte concentrations) should be kept as low as possible. Not only does this help maintain a higher relative concentration of electrolyte to analyte, but it serves to conserve valuable quantities of isotope, which are less easily recovered from both electrolyte and internal standards (Fe, Co) and minimize worker dose when working with higher specific-activity isotopes. To our knowledge, there are no published procedures for reprocessing residues containing electrochemical waste, and we suggest that the development of such strategies should be pursued.
(4) Special attention should be paid to the cleanliness and storage of electrodes, as they should be treated as contaminated even after cleaning and/or polishing. We suggest storing the WE, CE, and RE (sans frit) clean in a 15 mL falcon tube, which is capped and labeled with isotope identity. The frit can be stored in a small bottle or tall vial inside the electrolyte solution, also labeled with an isotope. Electrodes and frits used for TRU electrochemistry may have shorter-than-normal lifetimes due to contamination and should be treated as radioactive waste on disposal.

In performing electrochemical experiments on transuranic analytes, we have found it expedient to reduce the concentration of electrolyte to 0.05 M when using THF/TBABPh_4_ due to its lower solubility. This choice decreases the necessary amount of agitation of a solution containing transuranic materials. Since analyte concentrations are lowered to conserve isotope, this analyte solution still maintains a good electrolyte-to-analyte ratio. We note comparable behavior between the 0.1 and 0.05 M solutions of TBABPh_4_ in THF—the potentials of the metallocene standards are effectively the same, with similar ΔEp values. The only notable difference is that, surprisingly, the 0.05 M TBABPh_4_ solution boasts a slightly expanded anodic window, and can capture the complete, albeit distorted Fc^+/0^ couple (Figures S5, S9).

### Presentation of Data and SI

The ACS has recently set forth guidelines for the presentation of electrochemical data;^55^ however, there are a number of items we suggest including as they serve the wider community. Transparency in data and methods and the inclusion of instructive details are of the highest importance in ensuring that data can be understood, compared, and discussed across a wide audience. As such, we recommend the inclusion of a detailed electrochemical methods section which covers not only the standard criteria set forth by the ACS, but also explicitly describes the procedures used during the experiment. Critically, a discussion of all data treatments, including referencing, should be presented: not all referencing methods are equivalent. In discussing reversibility, care should be taken in the definitions of reversibility, quasireversibility, and irreversibility, and how they are treated in the text—the inclusion of Randles-Ševčík plots and scan-rate dependence voltammograms (which may be more illustrative when scan-rate normalized)^56^ should be standard when this is central to the analysis.

Other pertinent details include, but are not limited to (1) which scans/segments were used for analysis and graphics, (2) how much *iR* compensation was applied during the measurement, (3) if additional filters or collection parameters were employed, (4) the open circuit potential (OCP) of each analyte (more information on OCPs can be found in Dempsey’s guide^56^), and (5) cell volumes and any special conditions for electrodes or their use in the solution. We also suggest including a voltammogram of the reference compound used collected over the full electrochemical window as these data establish the potential of the reference redox couple in the absence of a reactive analyte. Additionally, if the blank electrolyte scans do reveal background impurities that cannot be removed (potentially in a TRU experiment), we encourage authors to include these data so that impurities in analyte CVs can be unambiguously identified. These should be presented alongside voltammograms of the analyte with an internal standard. Additionally, aspects of the cleaning, preparation, and storage of electrodes may be of interest to the community and can impact data collection and reproducibility. We also encourage the reporting of electrochemical windows for each solvent/electrolyte combination used under the experimental conditions. The availability of these data, especially as new combinations are employed, is critical in informing the field as the boundaries of redox chemistry are extended.

Our goal in preparing this Viewpoint is to provide a document that equips readers with the toolkit to collect high-quality data and share it in a way that is most helpful to the wider community. While there is a path to exploratory data that may not take the suggestions of this guide into strict consideration, attention should be given to how that data is used and interpreted in a manuscript and to how the authors want that data to be used in the future. As with any data type, if CV data are part of the basis of claims about oxidation state or key redox reactions, the data quality and methodologies should be well-documented.

## CONCLUSIONS AND OUTLOOK

The utilization of cyclic voltammetry as an analytical tool in the study of f-element complexes is growing rapidly and requires a robust toolkit to generate high-quality data. Many of these species require nonaqueous conditions and are reactive and/or radioactive. All of these are confounding elements in acquiring electrochemical data. Additionally, the use of pseudoreference electrodes in many nonaqueous systems can complicate analysis significantly. Navigating solvent and electrolyte incompatibilities, optimizing cell setups, and working with small solution volumes are nontrivial, and there are additional challenges in choosing solvents/electrolytes, referencing data, and discussing reversibility. All of these aspects shape how we collect, interpret, and report electrochemical data. This guide attempts to provide a toolkit and framework to develop a shared set of best practices for data collection and analysis.

## Supplementary Material



## Data Availability

CAD files for Teflon caps available at Figshare: 10.6084/m9.figshare.30456341

## Notes

Complete experimental and methodological details, electrochemical data, and additional tables (PDF)
