# Supplementary material for: A Guide to Nonaqueous Electrochemistry of f‑Element Complexes
Source: Inorg Chem. 2026 Feb 9;65(7):3758–70. doi: 10.1021/acs.inorgchem.5c05041 (PMC12933887; doi:10.1021/acs.inorgchem.5c05041)
Supplement: Supplementary file 1 [file ic5c05041_si_001.pdf]

Supporting Information for
A Guide to Non-Aqueous Electrochemistry of f-Element Complexes

Julie E. Niklas[†*], Matilda I. Duffy[†], and Henry S. La Pierre[†‡#*]

[†]School of Chemistry and Biochemistry, Georgia Institute of Technology, Atlanta, Georgia 30332-0400, United States

[‡]Nuclear and Radiological Engineering and Medical Physics Program, School of Mechanical Engineering, Georgia Institute of Technology, Atlanta, Georgia 30332-0400, United States

[#]Physical Sciences Division, Pacific Northwest National Laboratory, Richland, Washington 99352, United States

j.niklas@gatech.edu

la_pierre@gatech.edu

Contents

General Considerations 2

Detailed Procedures for Electrochemical Measurements 3

Experiment Setup and First Steps: 3

Data Collection: 3

Best practices and additional notes: 4

Additional Figures & Tables 6

References 23

General Considerations

Unless otherwise noted, all manipulations were performed with rigorous exclusion of oxygen and water using Schlenk techniques under UHP argon, or in a glovebox (Vigor) under a dinitrogen atmosphere (<0.1 ppm O_2/H_2O). The glovebox is equipped with two -35 ˚C freezers and a cold well. All glassware was stored in an oven overnight (>8h) at a temperature of ca. 160˚C prior to use.

Materials: All reagents were obtained from commercial suppliers and used as received except as described below. Molecular sieves were heated under vacuum at a temperature >250˚C for a minimum of 24 h. Alumina (Aluminum oxide, γ-phase Type N, 0.05 μm) was heated under vacuum at 200 °C for 48 h and stored in the glovebox. Tetrahydrofuran (THF), dichloromethane (DCM, CH_2Cl_2), and acetonitrile (ACN, CH_3CN) were purged with UHP-grade argon (Airgas) and passed through columns containing Q-5/alumina and molecular sieves in a commercial solvent purification system (JC Meyer Solvent Systems, Pure Process Technology), then stored over 10% v/v 3 Å molecular sieves in media bottles inside the glovebox. Fluorobenzene (PhF, C_6H_5F) and ortho-difluorobenzene (o-DFB, $C_6H_4F_2$) were degassed and fractionally distilled, then stored over 10% v/v 3 Å molecular sieves in media bottles inside the glovebox. Solvents were tested for residual water with a saturated solution of purple Na/benzophenone (ketyl) in THF prior to use. Ferrocene (Fc, $(C_5H_5)_2Fe$) and decamethylferrocene (DmFc, $(C_5(CH_3)_5)_2Fe$) were doubly sublimed and dried under vacuum prior to use. Cobaltocene (Cc, $(C_5H_5)_2Co$) was sublimed and dried under vacuum prior to use. Decamethylcobaltocene (DmCc, $C_5(CH_3)_5)_2Co$) was dried under vacuum prior to use; however, we note that DmCc does not have a long shelf life, and can be purified easily by recrystallization from pentane. Tetra-*n*-butlyammonium hexafluorophosphate ($TBAPF_6$) was doubly recrystallized from ethanol and dried under vacuum at 100 °C overnight prior to use. Tetra-*n*-butlyammonium tetraphenylborate ($TBABPh_4$) was triply recrystallized (2 times from 10% water in acetone, and once from 70:30 acetone:diethyl ether) and dried on a Schlenk line at 100 °C for 24 h. $TBABPh_4$ is a costly reagent to use in large quantities and the procedure is reproduced here for your convenience.[1] Complexes for which illustrative data is presented, Ce(NP^{t}Bu(pyrr)$_2$)$_4$, U(NP^{t}Bu(pyrr)$_2$)$_4$, Np(NP^{t}Bu(pyrr)$_2$)$_4$, and U(NP^{t}Bu(pip)$_2$)$_4$, were prepared as previously described.[1, 2]

Electrochemical data were measured using a Pine WaveDriver 20 Bipotentiostat/Galvanostat. Measurements were performed in a glovebox at ambient temperature (~25 °C, 298 K) under an atmosphere of N_2 using a standard three-electrode cell topped with a custom Teflon cap. A glassy carbon working electrode (3 mm diameter), a polished Ag wire reference electrode in a fritted capillary filled with the corresponding electrolyte solution, and a platinum wire counter (0.5 mm) electrode were used. The fritted capillary was stored in electrolyte solution in a sealed bottle when not in use, and the inner solution was replaced with fresh electrolyte solution prior to use. The glassy carbon and Ag wire electrodes were polished inside the glovebox before use and stored in the glovebox. Electrolyte solutions were prepared immediately prior to measurements. Measurements were made in positive feedback iR compensation mode using values determined by PF-RU (positive feedback- uncompensated resistance) measurements of the blank electrolyte solution. Six scans were collected and monitored during collection for any changes in electrochemical behavior across the scans. If the scans change over time, it is important to report all scans in the SI and note that the first scan is likely the 'true' behavior of the system, though additional interpretation using what is known about the system is necessary. In the data reported herein, all six scans were identical for each sample; scans 3 and 4 are reported. Voltammograms were referenced to the $Fc^{+/0}$ couple using the internal standard.

Detailed Procedures for Electrochemical Measurements

Preparation of Reference Electrode Frits:

A ceramic frit was dried in the oven overnight at 160 °C, then cycled into the glovebox. A small volume (~2 mL) of electrolyte solution was prepared at the desired concentration, and the frit was filled using a pipette then submerged in a bottle or tall vial containing the rest of the electrolyte solution, and capped. The frit was allowed to soak overnight, or for at least 3h prior to measurements. *If the frit is not completely soaked, the measurements will be unstable.*

Experiment Setup and First Steps*:*

Electrolyte solutions for the blank/reference vial and analyte vial were prepared immediately prior to measurement of each. Electrolyte (scaled to prepare 5 mL of solution of the desired concentration) was massed into each vial, and compound was added to one. Solvent (5 mL) was massed into the blank/reference vial, the vial was capped and agitated to completely dissolve the electrolyte. A small portion of this solution was used to refresh the solution inside the reference electrode frit- this is critical for solvent/electrolyte combinations which are not stable over long periods of time. The electrode cap was placed over the vial and the electrodes were inserted, then connected to the potentiostat. Uncompensated resistance measurements were taken (positive feedback) using initial steps of 100 Ω to determine the approximate solution resistance, then repeated using finer steps to determine the best value (prior to oscillations appearing in the measurement), and 95% of this value was used as the iR compensation for the measurements. *If the peak splitting does not seem adequate during measurements, this value can be increased slowly to 99% of the determined value.* Overcompensation should be avoided. This often sharpens the peak shape, and speak splitting values may become unrealistically small.

Data Collection:

Cyclic voltammograms were measured for the blank electrolyte solution and the full electrochemical window established. *For unstable measurements or oscillatory traces, stability filters and current response filters can be manually decreased until stable behavior is observed).* Ferrocene and decamethylferrocene (and/or any needed reference compounds) were added in very small amounts (no more than the tip of a microspatula). Cyclic voltammograms were again measured and the full electrochemical window established. *Note: the addition of ferrocene in some electrolyte/solvent mixtures can increase the positive upper edge of the window relative to the blank.* E_{pc}, E_{pa}, and $E_{1/2}$ values for both couples were noted. After a suitable reference voltammogram was recorded, the vial was capped and set aside for later. The electrodes were cleaned thoroughly—the CE and RE were rinsed with solvent and wiped clean, and the WE was rinsed, polished on a microvelvet pad with fine alumina slurry in hexanes, and rinsed and wiped again.

When collecting electrochemical window and reference data for this viewpoint, the needed iR compensation was established, a blank of the electrolyte solution showing the full electrochemical window was recorded, and then all four reference compounds were added (Fc; DmFc; DmCc; Cc) and a voltammogram was again recorded. In some cases, the electrochemical window was found to have expanded by 50-100 mV after addition of the compounds.

When collecting data on any other analyte:

Solvent (5 mL) was massed into the analyte vial, and the vial was capped and agitated until all components were completely dissolved. Electrodes were inserted through the electrode cap and connected to the potentiostat. The open circuit potential of the analyte solution was determined. Cyclic voltammetry experiments were performed on the analyte, taking care to expand potential ranges of each experiment slowly until couples are fully

captured, as to not irreparably over-reduce/oxidize the analyte or strip the solvent/electrolyte. At the end of the experiment, the internal standard was added directly to the analyte and a full scan remeasured. Electrodes were removed and cleaned again, and replaced in the blank/reference vial. Another CV of the reference compound(s) was collected and the E_{pc}, E_{pa}, and $E_{1/2}$ values noted and compared to the "before" scan to determine if/how much electrode drift occurred during the experiments. Electrodes were removed, cleaned, and stored. The frit was removed from the Ag wire and stored in its bottle, submerged and filled.

Best practices and additional notes:

- Collect CVs of your reference compounds (i.e. ferrocene) before and after the experiment as well one with it added as an internal standard in your analyte.
    - For analytes which are incompatible with the reference compound during the electrochemical experiment, an average of the reference compounds "before" and "after" $E_{1/2}$ values can be used, provided the amount of drift is small (ideally no more than 10-20 mV). Larger drifts may require manual corrections to appropriately reference. For a set of f-element imidophosphorane complexes, we have recorded the "before", "after", average, and internal reference values, finding that even with electrode drifts of over 50 mV, the average potential of the $Fc^{+/0}$ couple differed by 20 mV or less from the potential of the internally measured $Fc^{+/0}$ couple.
    - Pick the best reference compound to add to your analyte- it should not overlap with existing features. Noting the positions of your references beforehand assists in determining which you should use. Measuring two reference compounds also establishes a relationship between the two, making it possible to reference to ferrocene by proxy of the other if ferrocene overlaps or is incompatible with your analyte.
- It is good practice, especially for reactive and/or potentially unstable species, to start your first scan with a small window and at mild potentials, and gradually increase the scan range. Once you have determined the species is stable in a certain range, it may be a good idea to collect scan rate dependence and any other desired data, then full window scans should be conducted. If the analyte is not stable at a certain potential, or after a redox event, the sample may need to be prepared again to collect the necessary data, especially if the bulk solution is impacted. This is not uncommon with very high- or low- oxidation state species.
- If multiple features are observed, scan each individually to determine if there is a relationship between it and any other feature in the full window.
- Scan rate dependence measurements are best collected on each feature in isolation as well as on the full window.
- If new features appear after a certain scan, perturb the solution and remeasure to determine if the bulk solution has been impacted. If they persist, cleaning of the WE surface may be required.
- Solution may be perturbed between experiments, but do not stir or move the solution during measurement.
- For measurements of radioactive analytes, the glovebox should be maintained with the lowest levels of contamination possible. Using a disposable cell is one way to help achieve

this. It is also best practice to use designated cells and electrodes for different isotopes, as separation of isotopes can be problematic.

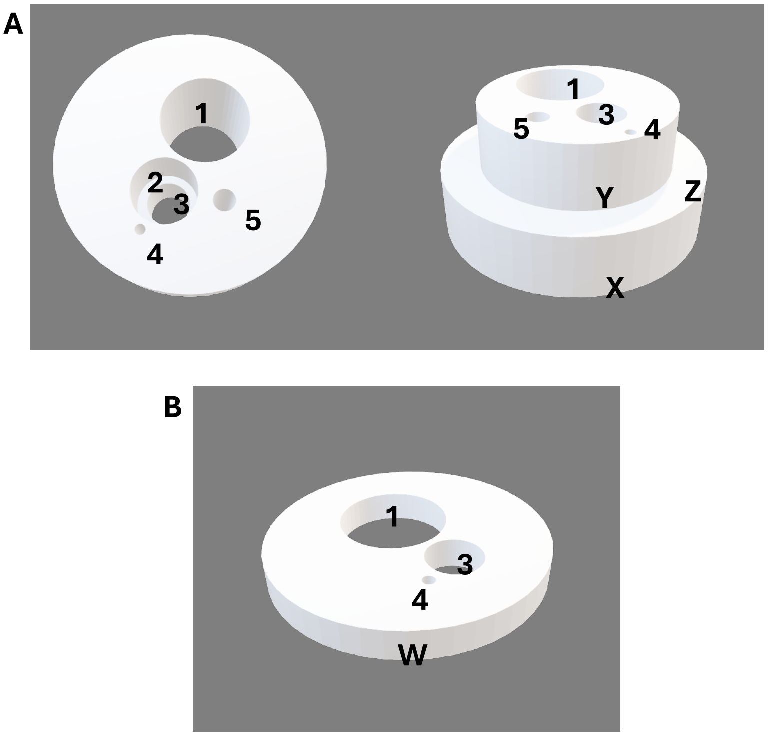

Figure S1. Depictions of Teflon caps used for (A) standard 20-mL vials and (B) 5-mL conical high-recovery vials. B is intended to seat underneath an open-hole threaded cap. CAD files are provided separately. 1: 7.00 mm; 2: 5.1 mm x 5 mm H; 3: 3.60 mm ; 4: 0.80 mm; 5: 1.70 mm; X: 20.64 mm OD x 5.00 mm H; Y: 15.00 mm OD x 5.00 mm H; Z: 2.82 mm W; W: 10.00 mm x 2.00 mm. CAD files are available as additional supporting information.

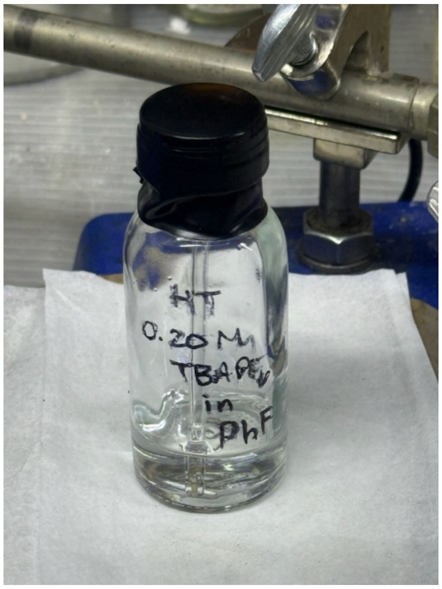

Photo S1. Storage of ceramic-tipped frit for RE in electrolyte solution.

Table S1. Potentials (V) of the $Fc^{+/0}$ couple vs $Ag^{+/0}$ in various solvent/electrolyte combinations.

	THF	PhF	oDFB	ACN	DCM
**$TBAPF_6$**	1.13	1.26	0.94	0.76	0.81
**$TBABPh_4$**	~1.16*	-	-	0.89	1.01

Cell conditions: GC WE, Pt wire CE, bare Ag^0 wire, fritted RE. All electrolyte concentrations 0.10 M, with exception of DCM/$TBABPh_4$ (0.05 M). *Only an Ep_c value is available- value is estimated from $DmFc^{+/0}$ couple and adjusted by 0.50 V.

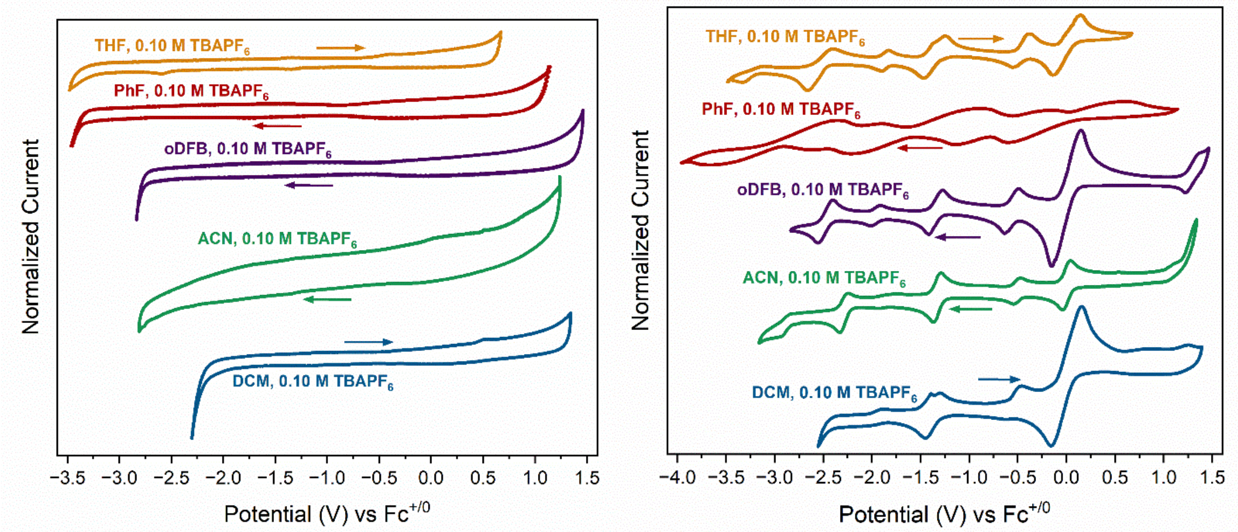

Figure S2. Comparison of a selection of solvents with $TBAPF_6$ electrolyte with no internal references (left) and with Fc, DmFc, Cc, and DmCc (right).

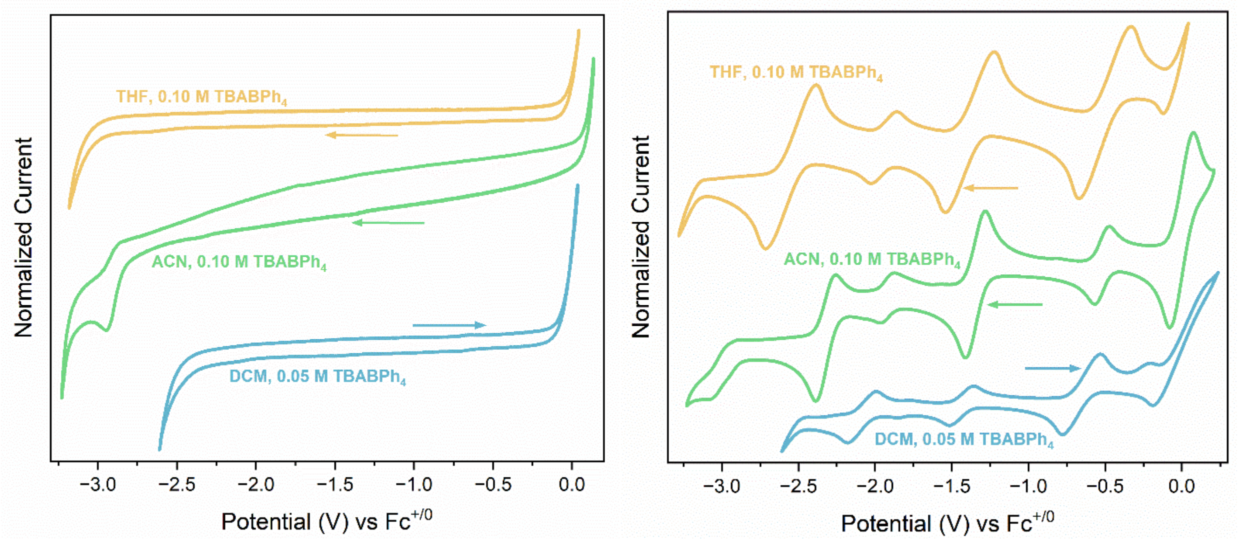

Figure S3. Comparison of a selection of solvents with $TBABPh_4$ electrolyte with no internal references (left) and with Fc, DmFc, Cc, and DmCc (right).

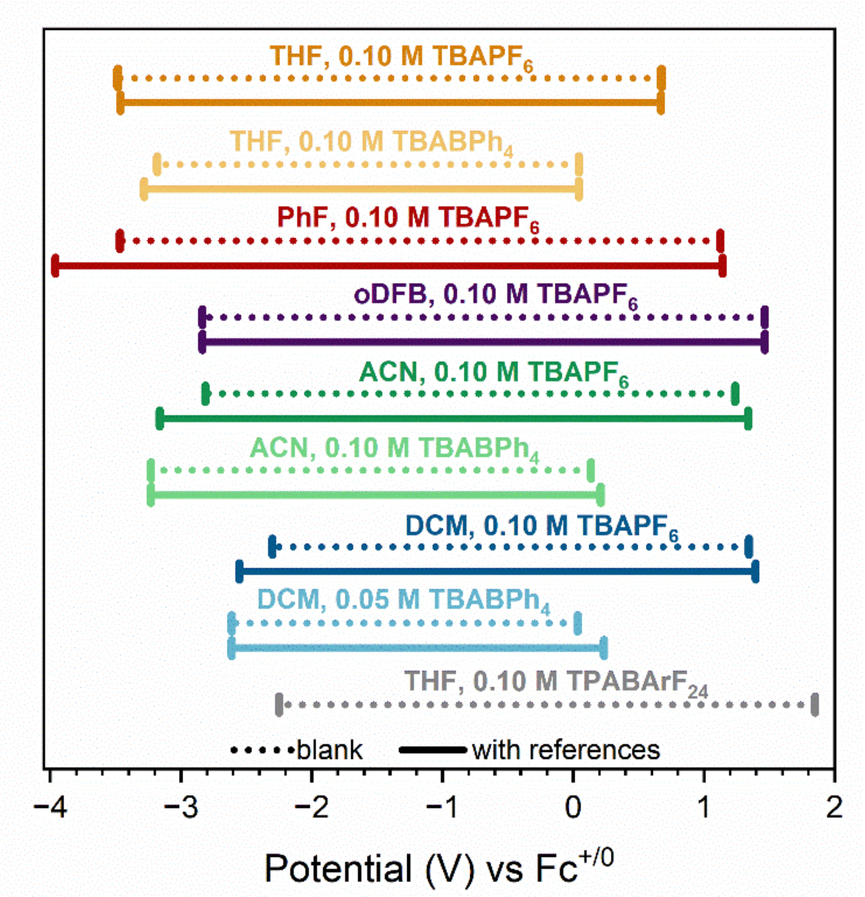

Figure S4. Scan windows for various solvent and electrolyte combinations. Dotted lines indicate the absence of internal references, solid lines indicate that solutions contained Fc, DmFc, Cc, and DmCc. THF, 0.10 M $TPABArF_{24}$ window previously established.[3]

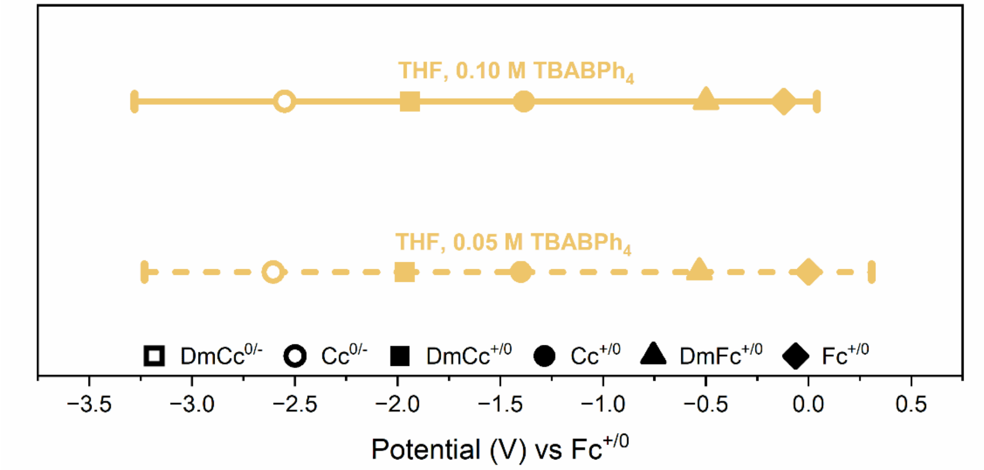

Figure S5. Scan windows and $E_{1/2}$ values of metallocene references for two different concentrations of $TBABPh_4$ in THF. The $Fc^{+/0}$ in the 0.10 M $TBABPh_4$ sample is an Ep_c value.

Table S2. Potentials (V vs $Fc^{+/0}$), Ep, and ΔEp values for all internal standard metallocenes measured in various solvent/electrolyte combinations.

		THF $TBAPF_6$	THF $TBABPh_4$	PhF $TBAPF_6$	oDFB $TBAPF_6$	ACN $TBAPF_6$	ACN $TBABPh_4$	DCM $TBAPF_6$	DCM $TBABPh_4$
**$DmCc^{0/-}$**	Ep_c	-3.33	--	--	--	-3.00	-3.07	--	--
	Ep_a	-3.12	--	--	--	-2.84	-2.90	--	--
	$E_{1/2}$	-3.23	--	--	--	-2.92	-2.99	--	--
	ΔEp	0.22	--	--	--	0.16	0.17	--	--
**$Cc^{0/-}$**	Ep_c	-2.67	-2.72	-3.12	-2.55	-2.33	-2.39	--	--
	Ep_a	-2.39	-2.38	-2.29	-2.40	-2.24	-2.26	--	--
	$E_{1/2}$	-2.53	-2.55	-2.70	-2.47	-2.29	-2.32	--	--
	ΔEp	0.28	0.33	0.83	0.15	0.09	0.13	--	--
**$DmCc^{+/0}$**	Ep_c	-1.91	-2.03	-2.64	-2.01	-1.94	-1.97	-2.03	-2.18
	Ep_a	-1.82	-1.85	-1.89	-1.91	-1.86	-1.88	-1.90	-1.99
	$E_{1/2}$	-1.87	-1.94	-2.26	-1.96	-1.90	-1.92	-1.97	-2.08
	ΔEp	0.09	0.17	0.76	0.10	0.07	0.09	0.13	0.18
**$Cc^{+/0}$**	Ep_c	-1.47	-1.55	-1.83	-1.42	-1.36	-1.41	-1.46	-1.52
	Ep_a	-1.24	-1.22	-1.01	-1.27	-1.29	-1.29	-1.36	-1.36
	$E_{1/2}$	-1.36	-1.38	-1.42	-1.35	-1.33	-1.35	-1.41	-1.44
	ΔEp	0.23	0.32	0.82	0.15	0.07	0.12	0.10	0.16
**$DmFc^{+/0}$**	Ep_c	-0.56	-0.68	-0.85	-0.63	-0.53	-0.57	-0.69	-0.78
	Ep_a	-0.38	-0.32	-0.29	-0.49	-0.47	-0.48	-0.56	-0.53
	$E_{1/2}$	-0.47	-0.50	-0.57	-0.56	-0.50	-0.52	-0.63	-0.65
	ΔEp	0.18	0.35	0.56	0.14	0.06	0.09	0.13	0.25
**$Fc^{+/0}$**	Ep_c	-0.14	-0.12	-0.36	-0.15	-0.03	-0.08	-0.16	-0.19
	Ep_a	0.15	--	0.37	0.15	0.04	0.08	0.16	0.19
	$E_{1/2}$	0.00	--	0.00	0.00	0.00	0.00	0.00	0.00
	ΔEp	0.29	--	0.73	0.30	0.07	0.15	0.32	0.38
**$DmFc^{2+/+}$**	Ep_c	--	--	1.10	1.22	1.00	--	1.01	--
	Ep_a	--	--	--	1.40	1.20	--	1.26	--
	$E_{1/2}$	--	--	--	1.31	1.10	--	1.13	--
	ΔEp	--	--	--	0.18	0.20	--	0.25	--

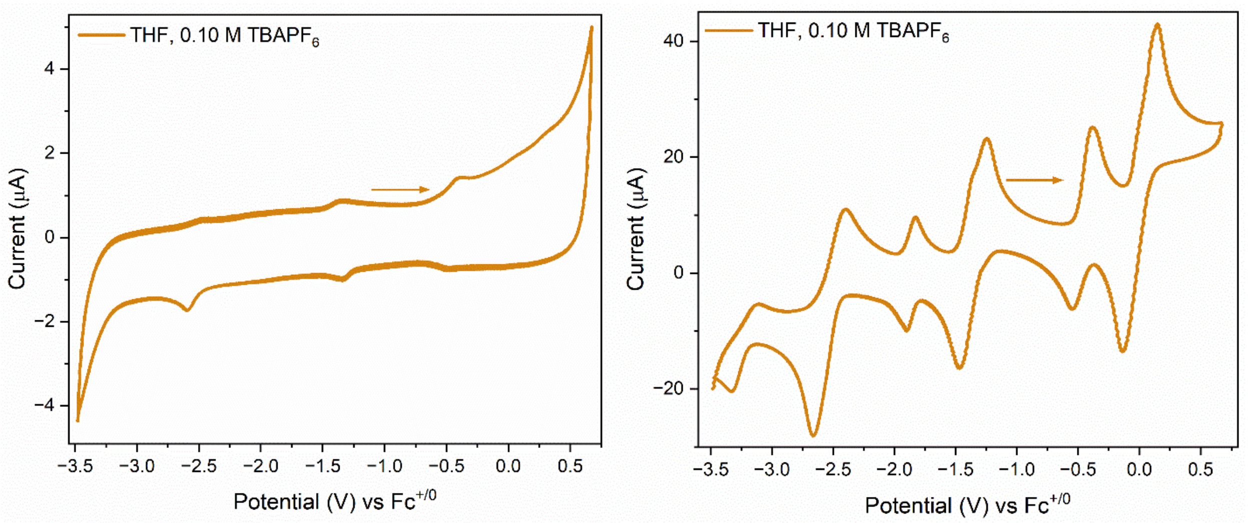

Figure S6. 0.10 M $TBAPF_6$ in THF with no internal references (left) and Fc, DmFc, Cc, and DmCc (right). WE: GC; CE: Pt wire; RE: fritted Ag^0 wire; iR compensation of 665 Ω, stability and current response filters were used.

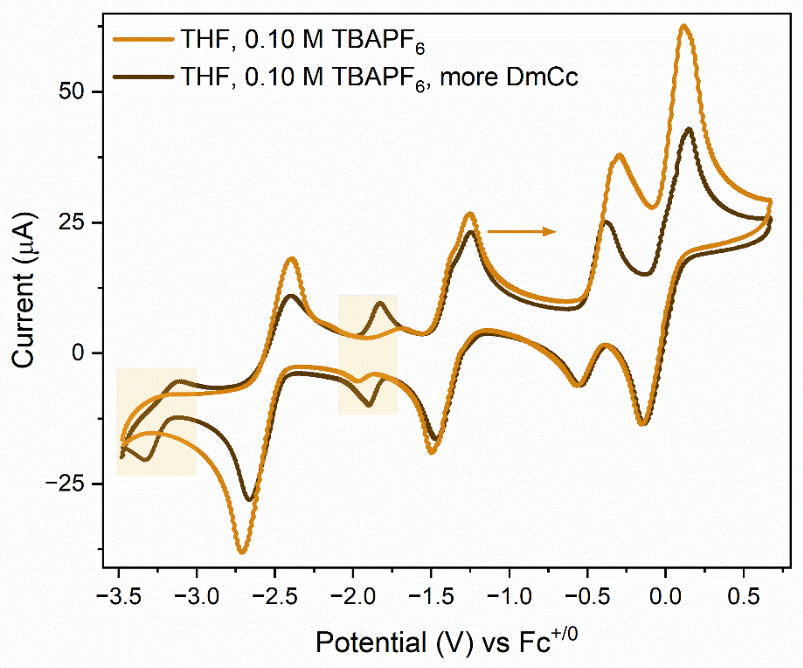

Figure S7. 0.10 M $TBAPF_6$ in THF with Fc, DmFc, Cc, and DmCc (light trace) and additional DmCc added (dark trace). The features related to DmCc are highlighted. The arrow demonstrating direction of the scan applies to both traces. WE: GC; CE: Pt wire; RE: fritted Ag^0 wire; iR compensation of 665 Ω, stability and current response filters were used.

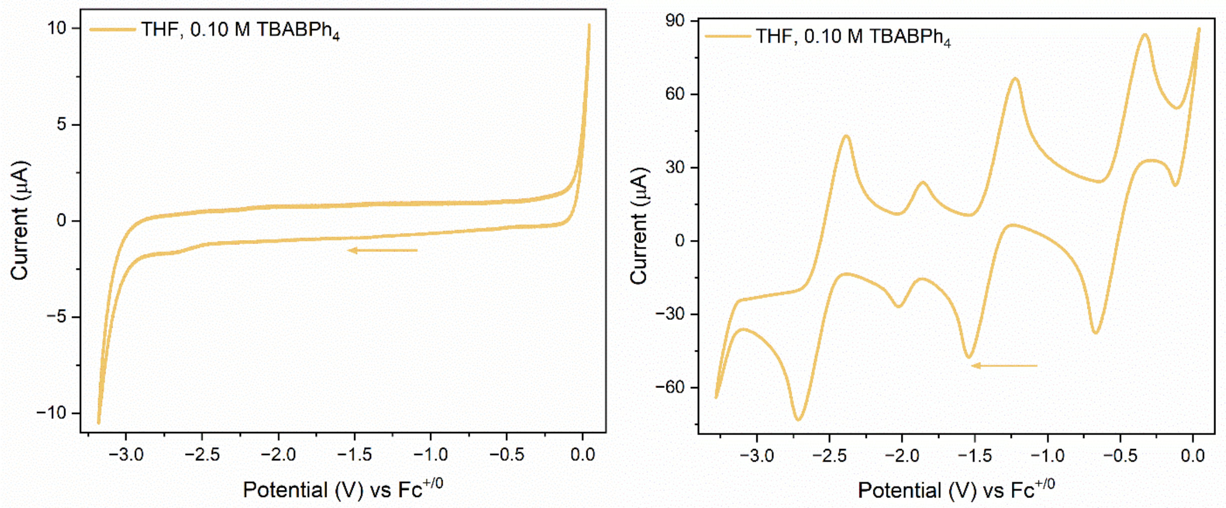

Figure S8. 0.10 M $TBABPh_4$ in THF with no internal references (left) and Fc, DmFc, Cc, and DmCc (right). WE: GC; CE: Pt wire; RE: fritted Ag^0 wire; iR compensation of 40 Ω.

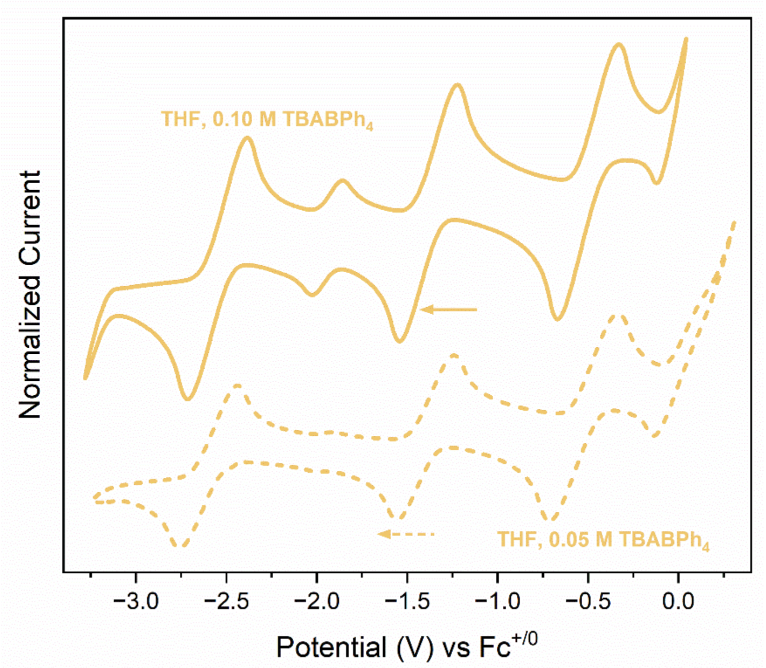

Figure S9. 0.10 M $TBABPh_4$ in THF (solid trace) and 0.05 M $TBABPh_4$ in THF (dashed trace) with Fc, DmFc, Cc, and DmCc. WE: GC; CE: Pt wire; RE: fritted Ag^0 wire; iR compensation of 40 Ω for 0.10 M $TBABPh_4$ sample. WE: GC; CE: Pt wire; RE: fritted Ag^0 wire; iR compensation of 285 Ω for 0.05 M $TBABPh_4$ sample.

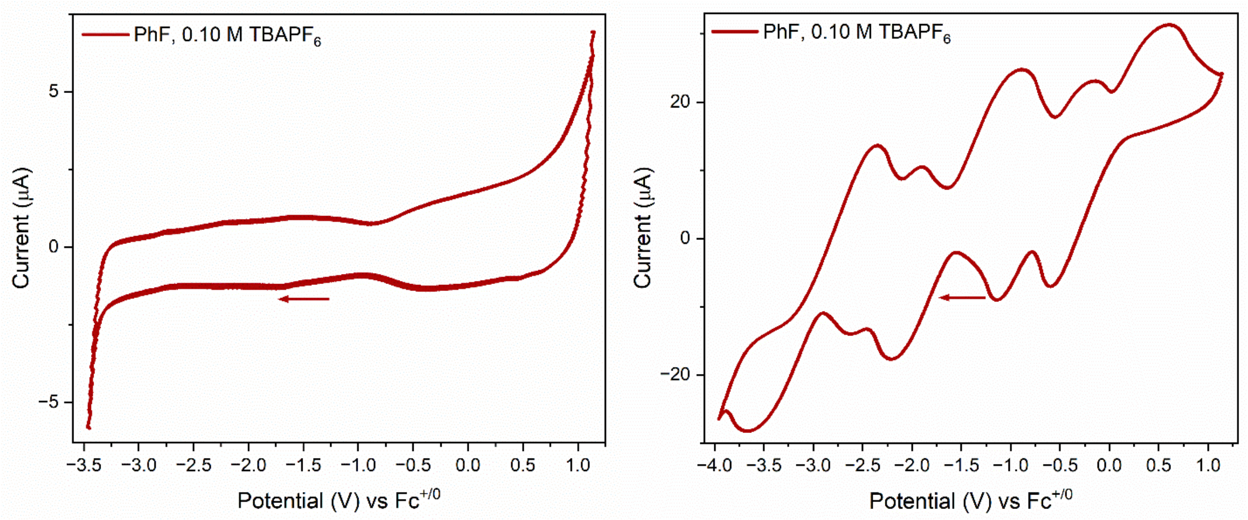

Figure S10. 0.10 M $TBAPF_6$ in PhF with no internal references (left) and with Fc, DmFc, Cc, and DmCc (right). WE: GC; CE: Pt wire; RE: fritted Ag^0 wire; iR compensation of 1900 Ω, stability, current, and potential response filters were used.

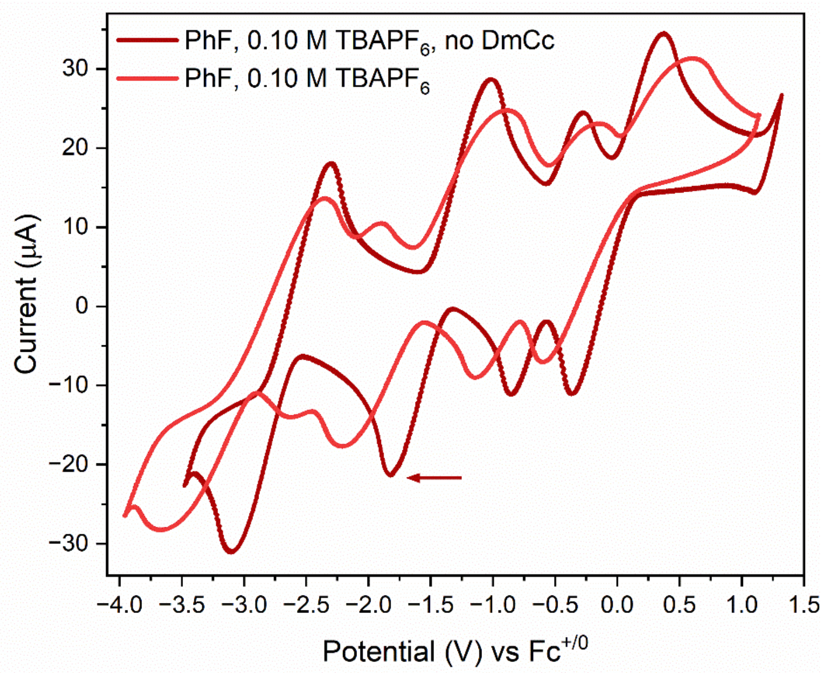

Figure S11. 0.10 M $TBAPF_6$ in PhF with Fc, DmFc, and Cc (dark trace) and with DmCc added (light trace). The effect of DmCc on the appearance of the trace is visualized. The $E_{1/2}$ for all references is the same in both traces, with the exception of the $Cc^{0/-}$ feature, which is broadened due to the overlap of this feature with the $DmCc^{0/-}$ feature (See Table S2). The arrow demonstrating direction of the scan applies to both traces. WE: GC; CE: Pt wire; RE: fritted Ag^0 wire; iR compensation of 1900 Ω, stability, current, and potential response filters were used.

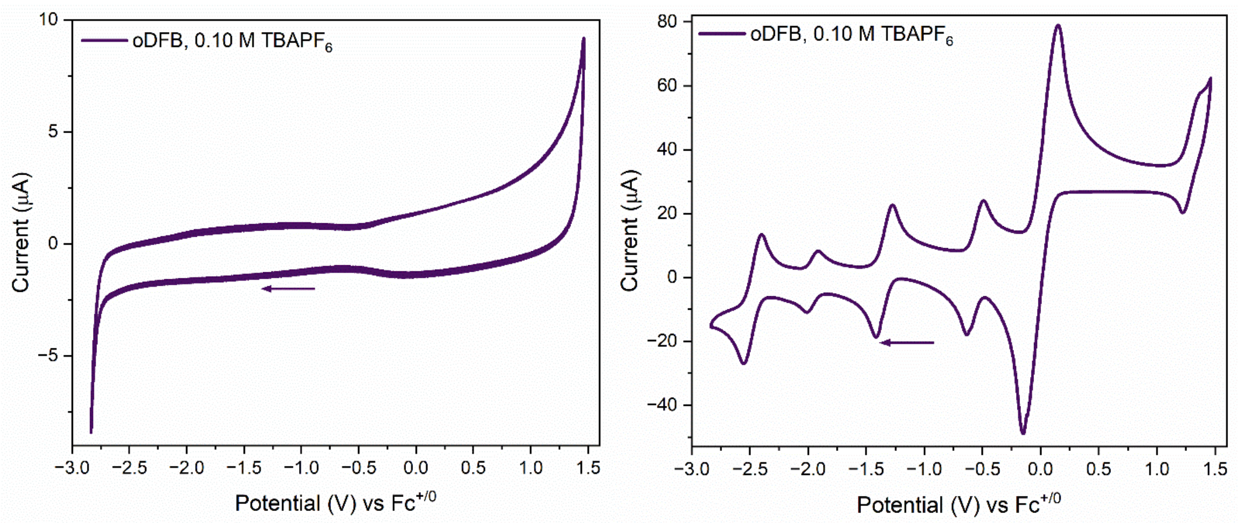

Figure S12. 0.10 M $TBAPF_6$ in oDFB with no internal references (left) and with Fc, DmFc, Cc, and DmCc (right). WE: GC; CE: Pt wire; RE: fritted Ag^0 wire; iR compensation of 70 Ω, stability and current response filters were used.

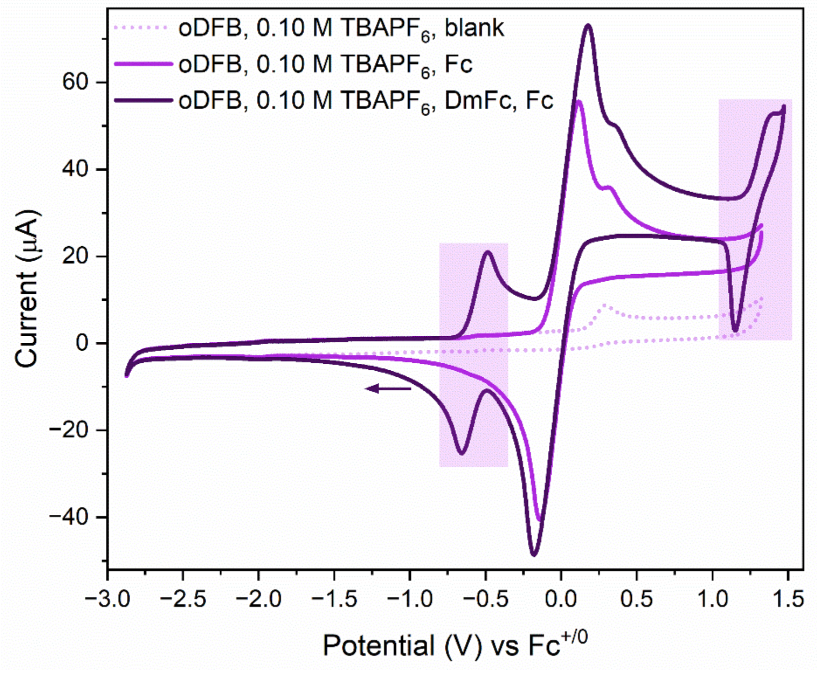

Figure S13. 0.10 M $TBAPF_6$ in oDFB with no internal references (dotted trace), with only Fc (light trace), and with Fc and DmFc (dark trace). The features related to DmFc are highlighted. The feature in the blank is attributed to solvent impurity. The arrow demonstrating direction of the scan applies to all three traces. WE: GC; CE: Pt wire; RE: fritted Ag^0 wire; iR compensation of 70 Ω, stability and current response filters were used.

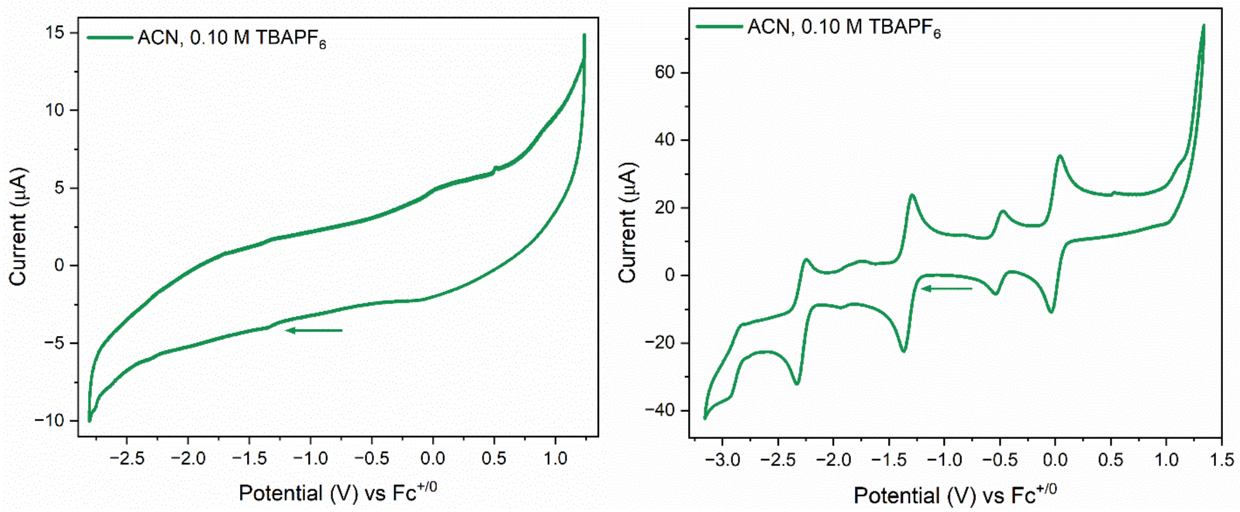

Figure S14. 0.10 M $TBAPF_6$ in ACN with no internal references (left) and with Fc, DmFc, Cc, and DmCc (right). WE: GC; CE: Pt wire; RE: fritted Ag^0 wire.

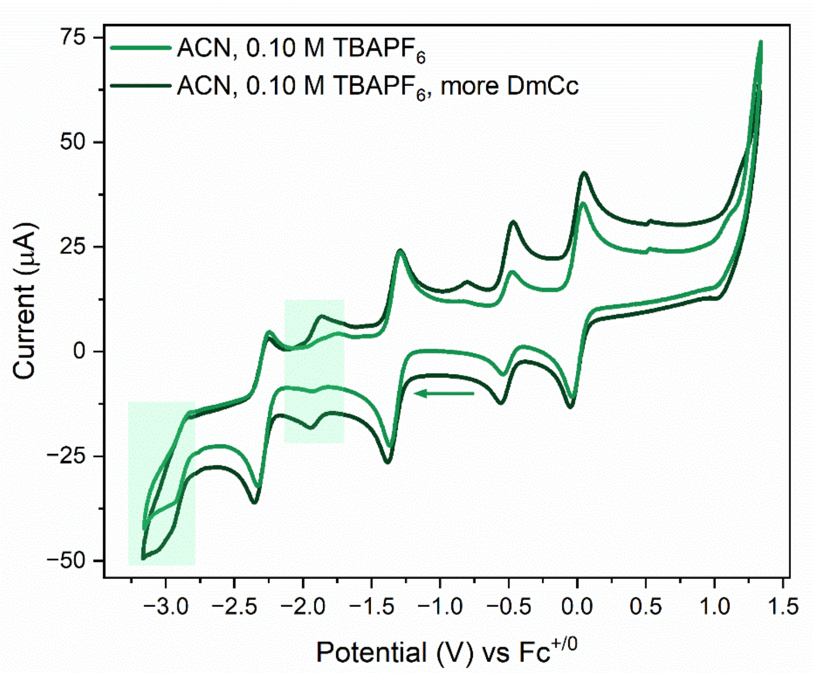

Figure S15. 0.10 M $TBAPF_6$ in ACN with Fc, DmFc, Cc, and DmCc (light trace) and additional DmCc added (dark trace). The features related to DmCc are highlighted. The arrow demonstrating direction of the scan applies to both traces. WE: GC; CE: Pt wire; RE: fritted Ag^0 wire.

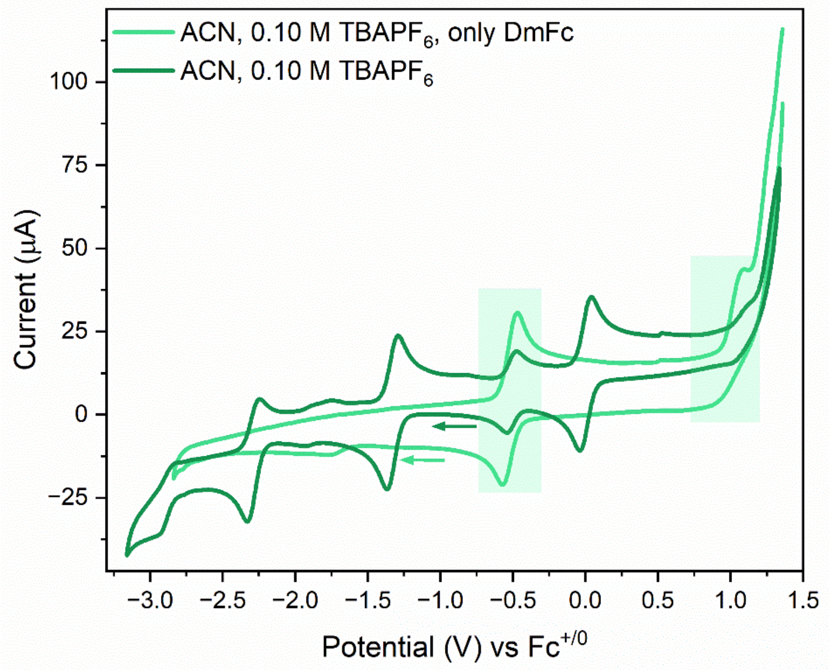

Figure S16. 0.10 M $TBAPF_6$ in ACN with only DmFc (light trace) and with Fc, DmFc, Cc, and DmCc (dark trace). The features related to DmFc are highlighted. WE: GC; CE: Pt wire; RE: fritted Ag^0 wire.

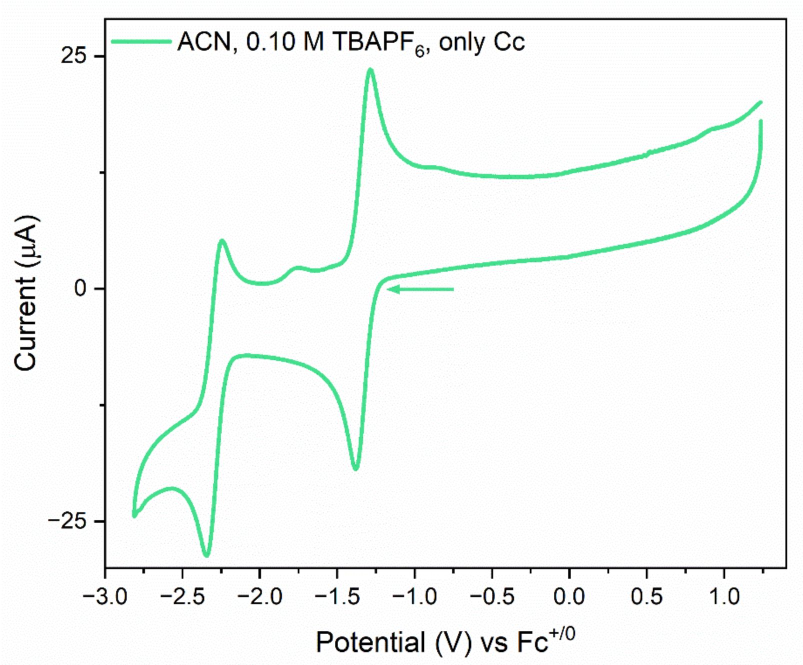

Figure S17. 0.10 M $TBAPF_6$ in ACN with only Cc. WE: GC; CE: Pt wire; RE: fritted Ag^0 wire.

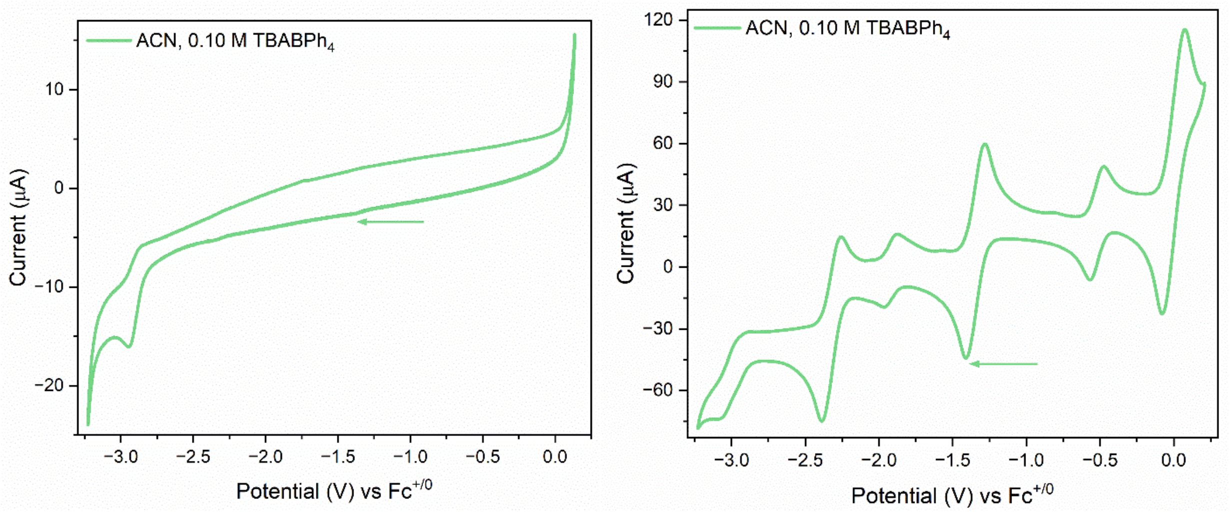

Figure S18. 0.10 M $TBABPh_4$ in ACN with no internal references (left) and with Fc, DmFc, Cc, and DmCc (right). WE: GC; CE: Pt wire; RE: fritted Ag^0 wire.

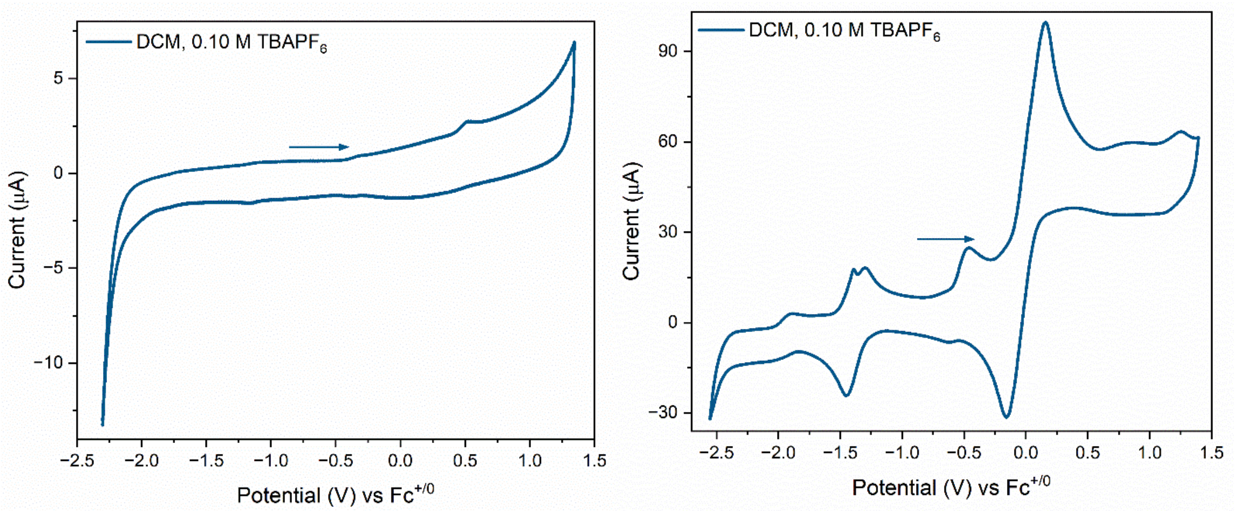

Figure S19. 0.10 M $TBAPF_6$ in DCM with no internal references (left) and with Fc, DmFc, Cc, and DmCc (right). WE: GC; CE: Pt wire; RE: fritted Ag^0 wire.

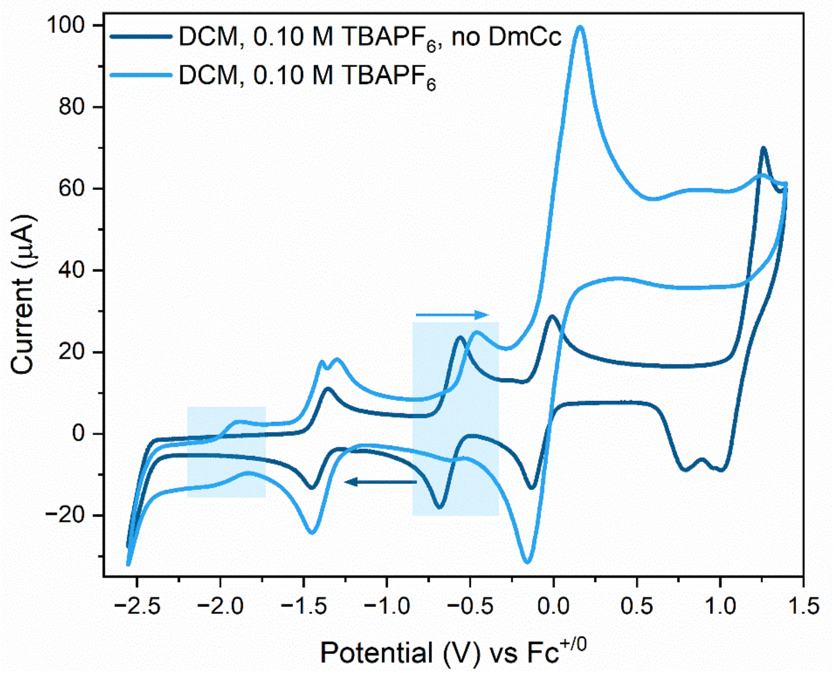

Figure S20. 0.10 M $TBAPF_6$ in DCM with Fc, DmFc, and Cc (dark trace) and with Fc, DmFc, Cc, and DmCc (light trace). The features related to DmCc are highlighted. Reactivity of DmCc with DCM yields a Cp*Co(η^4-$C_5Me_5CH_2Cl$)$^{+/0}$ feature at -0.46 V vs $Fc^{+/0}$, which overlaps with the $DmFc^{+/0}$ feature.[4] WE: GC; CE: Pt wire; RE: fritted Ag^0 wire.

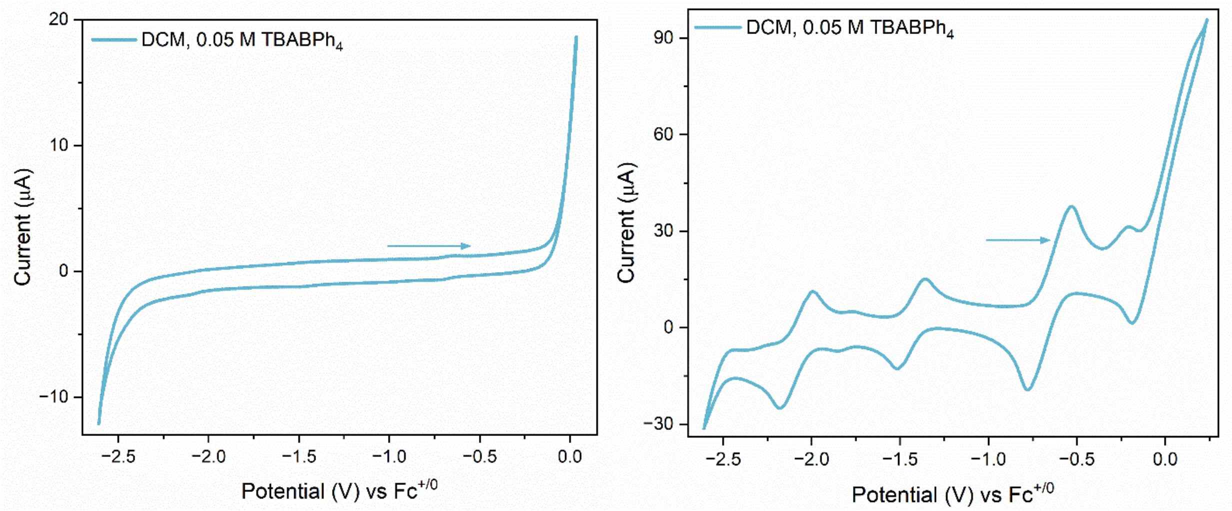

Figure S21. 0.05 M $TBABPh_4$ in DCM with no internal references (left) and with Fc, DmFc, Cc, and DmCc (right). WE: GC; CE: Pt wire; RE: fritted Ag^0 wire.

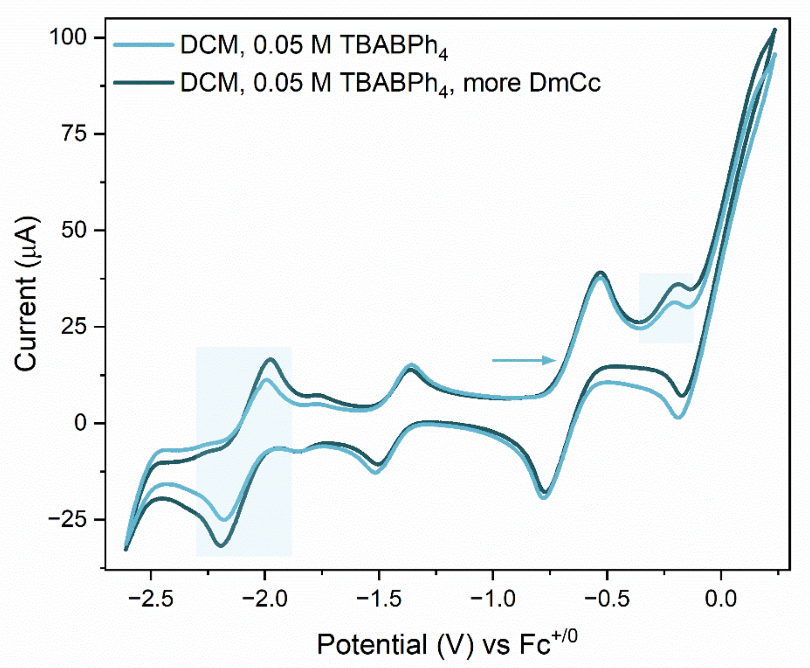

Figure S22. 0.05 M $TBABPh_4$ in DCM with Fc, DmFc, Cc and DmCc (light trace) and additional DmCc (dark trace). The features related to DmCc are highlighted. Reactivity of DmCc with DCM yields a Cp*Co(η^4-$C_5Me_5CH_2Cl$)$^{+/0}$ feature at -0.21 V vs $Fc^{+/0}$. [4] WE: GC; CE: Pt wire; RE: fritted Ag^0 wire.

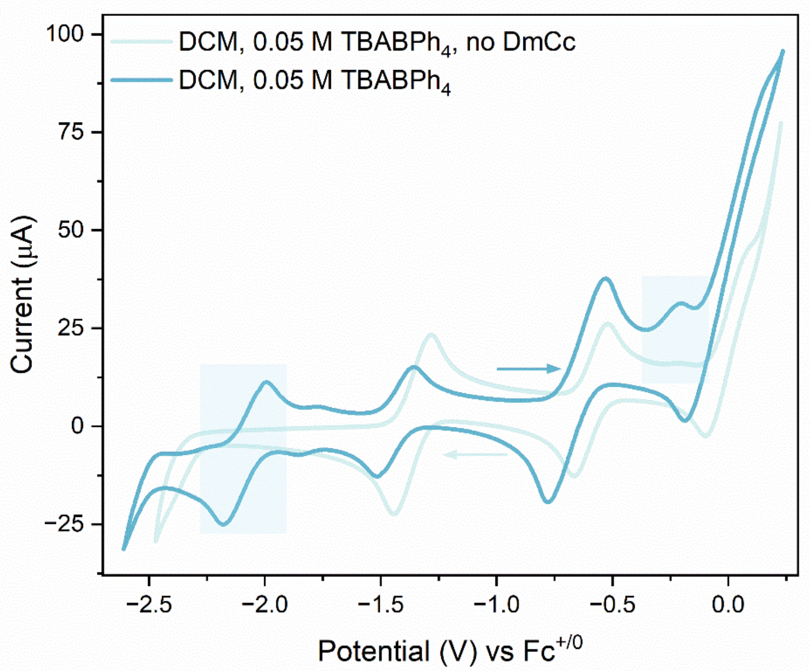

Figure S23. 0.05 M $TBABPh_4$ in DCM with Fc, DmFc, and Cc (light trace) and with Fc, DmFc, Cc, and DmCc (dark trace). The features related to DmCc are highlighted. Reactivity of DmCc with DCM yields a Cp*Co(η^4-$C_5Me_5CH_2Cl$)$^{+/0}$ feature at -0.21 V vs $Fc^{+/0}$. [4] WE: GC; CE: Pt wire; RE: fritted Ag^0 wire.

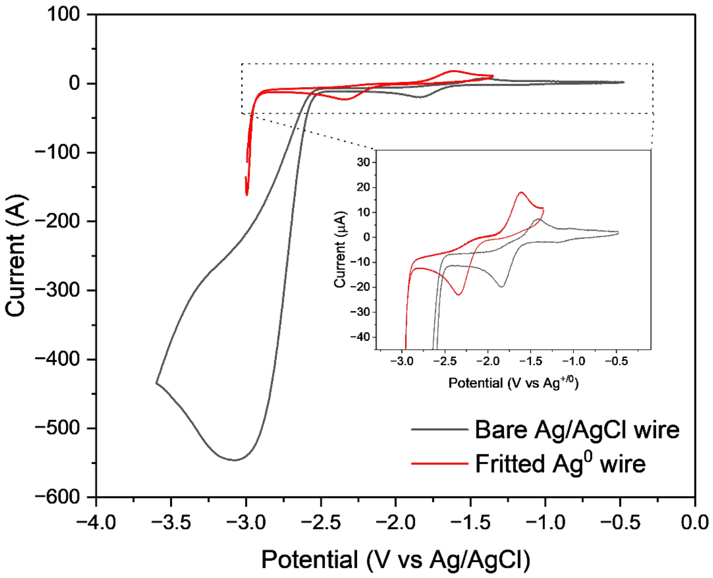

Figure S24. Comparison of reference electrode conditions for Ce(NP^{t}Bu(pyrr)$_2$)$_4$ at approx. 3 mM in THF with 0.05 M TBABPh$_4$ supporting electrolyte. WE: GC; CE: Pt wire; RE: fritted Ag^0 wire or bare Ag/AgCl wire. A shift in the effective $E_{1/2}$ of over 400 mV is noted between methods.

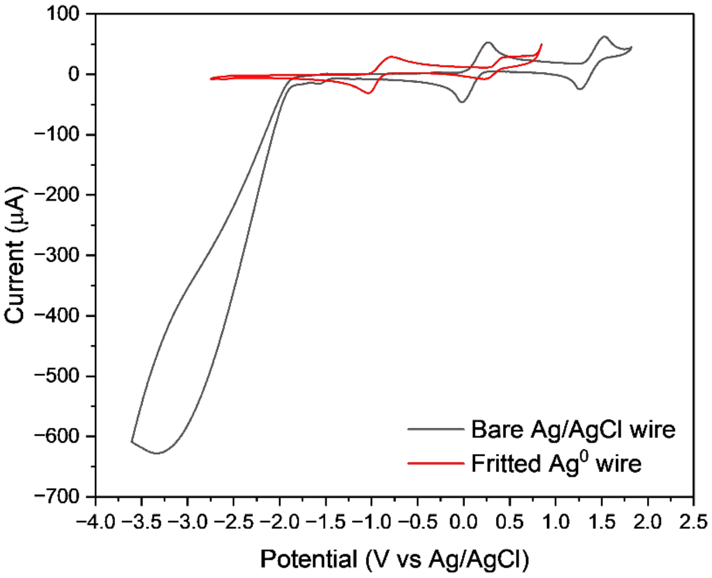

Figure S25. Comparison of reference electrode conditions for U(NP^{t}Bu(pyrr)$_2$)$_4$ at approx. 3 mM in THF with 0.05 M TBABPh$_4$ supporting electrolyte. WE: GC; CE: Pt wire; RE: fritted Ag^0 wire or bare Ag/AgCl wire. A shift in the effective $E_{1/2}$ of over 1 V is noted between methods.

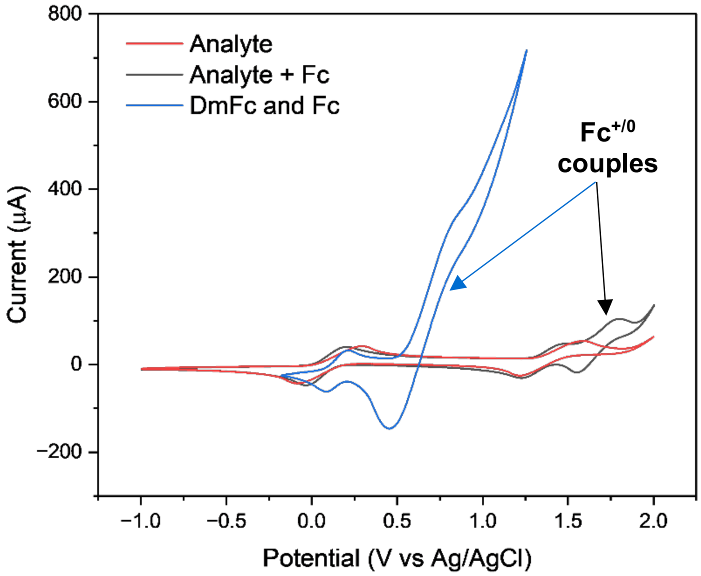

Figure S26. Voltammograms of U(NP^{t}Bu(pyrr)$_2$)$_4$ (red: compound only; black: with ferrocene added) at approx. 3 mM in THF with 0.05 M TBABPh$_4$ supporting electrolyte. WE: GC; CE: Pt wire; RE: bare Ag/AgCl wire. Blue trace shows ferrocene and decamethylferrocene recorded under the same conditions following the experiment. A shift in the potential of the ferrocene couple of over 1 V (or reactivity) is noted, with small shifts (~20 mV) in the $U^{5+/4+}$ and $U^{6+/5+}$ couples.

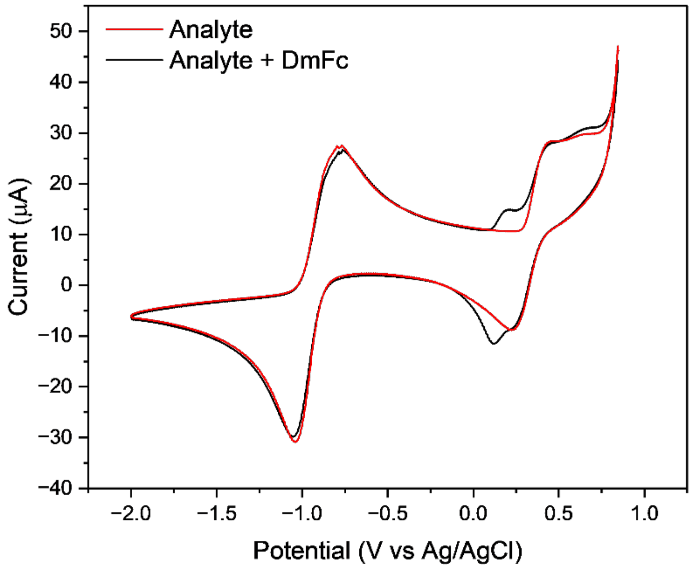

Figure S27. Voltammograms of U(NP^{t}Bu(pyrr)$_2$)$_4$ (red: compound only; black: with decamethylferrocene added) at approx. 3 mM in THF with 0.05 M TBABPh$_4$ supporting electrolyte. WE: GC; CE: Pt wire; RE: fritted Ag^0 wire. No shifts in the potentials of decamethylferrocene or the U couples are observed, and there is no apparent reactivity.

Table S3. Comparison of ferrocene and decamethylferrocene potentials measured externally and internally in 0.05 M $TBABPh_4$ in THF for a variety of Ln and An imidophosphorane compounds using and **fritted** Ag^0 RE, GC WE, and Pt wire CE. In some cases the Fc couple could not be observed. Potentials are in V, *vs* Ag/AgCl.

		Before				After				Average				Internal			
		Ep_a	Ep_c	$E_{1/2}$	ΔE	Ep_a	Ep_c	$E_{1/2}$	ΔE	Ep_a	Ep_c	$E_{1/2}$	ΔE	Ep_a	Ep_c	$E_{1/2}$	ΔE
	Fc	0.68	0.54	0.61	0.14	0.72	0.66	0.69	0.06			0.65	0.10				
	DmFc	0.17	0.08	0.13	0.09	0.24	0.19	0.22	0.05			0.17	0.07	0.19	0.12	0.16	0.07
**Np(NP^{t}Bu(pyrr)2)4**	DmFc v Fc			-0.49				-0.48				-0.48					
	Shift Fc					0.04	0.12	0.08						Diff:		0.02	
	Shift DmFc					0.07	0.11	0.09									

		Before				After				Average				Internal			
		Ep_a	Ep_c	$E_{1/2}$	ΔE	Ep_a	Ep_c	$E_{1/2}$	ΔE	Ep_a	Ep_c	$E_{1/2}$	ΔE	Ep_a	Ep_c	$E_{1/2}$	ΔE
	Fc	0.73	0.48	0.61	0.25	0.65	0.52	0.59	0.13	0.69	0.50	0.60	0.19				
	DmFc	0.31	-0.15	0.08	0.46	0.19	0.07	0.13	0.12	0.25	-0.04	0.11	0.29	0.16	0.08	0.12	0.08
**Ce(NP^{t}Bu(pyrr)2)4**	DmFc v Fc			-0.53				-0.46				-0.49					
	Shift Fc					-0.08	0.04	-0.02						Diff:		-0.02	
	Shift DmFc					-0.12	0.22	0.05									

		Before				After				Average				Internal			
		Ep_a	Ep_c	$E_{1/2}$	ΔE	Ep_a	Ep_c	$E_{1/2}$	ΔE	Ep_a	Ep_c	$E_{1/2}$	ΔE	Ep_a	Ep_c	$E_{1/2}$	ΔE
	Fc	0.65	0.52	0.59	0.13	0.77	0.50	0.64	0.27	0.71	0.51	0.61	0.20				
	DmFc	0.19	0.07	0.13	0.12	0.25	0.04	0.15	0.21	0.22	0.06	0.14	0.17	0.19	0.12	0.16	0.07
**U(NP^{t}Bu(pyrr)2)4**	DmFc v Fc			-0.46				-0.49				-0.47					
	Shift Fc					0.12	-0.02	0.05						Diff:		0.02	
	Shift DmFc					0.06	-0.03	0.02									

		Before				After				Average				Internal			
		Ep_a	Ep_c	$E_{1/2}$	ΔE	Ep_a	Ep_c	$E_{1/2}$	ΔE	Ep_a	Ep_c	$E_{1/2}$	ΔE	Ep_a	Ep_c	$E_{1/2}$	ΔE
	Fc	0.77	0.50	0.64	0.27	0.79	0.49	0.64	0.30	0.78	0.50	0.64	0.29				
	DmFc	0.25	0.04	0.15	0.21	0.25	0.03	0.14	0.22	0.25	0.04	0.14	0.22	0.18	0.09	0.14	0.09
**U(NP^{t}Bu(pip)2)4**	DmFc v Fc			-0.49				-0.50				-0.50					
	Shift Fc					0.02	-0.01	0.01						Diff:		-0.01	
	Shift DmFc					0.00	-0.01	0.00									

Table S4. Comparison of ferrocene and decamethylferrocene potentials measured externally and internally in 0.05 M $TBABPh_4$ in THF for a variety of Ln and An imidophosphorane compounds using and **unfritted** Ag/AgCl RE, GC WE, and Pt wire CE. In some cases the Fc couple could not be observed. Potentials are in V, *vs* Ag/AgCl.

		Before				After				Average				Internal			
		Ep_a	Ep_c	$E_{1/2}$	ΔE	Ep_a	Ep_c	$E_{1/2}$	ΔE	Ep_a	Ep_c	$E_{1/2}$	ΔE	Ep_a	Ep_c	$E_{1/2}$	ΔE
	Fc	0.75	0.62	0.68	0.13	0.97	0.78	0.88	0.19	0.86	0.70	0.78	0.16	/	/		
	DmFc	0.23	0.16	0.20	0.07	0.43	0.33	0.38	0.10	0.33	0.25	0.29	0.09	0.79	0.69	0.74	0.10
**Np(NP^{t}Bu(pyrr)2)4**	DmFc v Fc			-0.49				-0.50				-0.49					
	Shift Fc					0.22	0.16	0.19						Diff:		0.45	
	Shift DmFc					0.20	0.17	0.19									

		Before				After				Average				Internal			
		Ep_a	Ep_c	$E_{1/2}$	ΔE	Ep_a	Ep_c	$E_{1/2}$	ΔE	Ep_a	Ep_c	$E_{1/2}$	ΔE	Ep_a	Ep_c	$E_{1/2}$	ΔE
	Fc	0.92	0.72	0.82	0.20	0.90	0.47	0.69	0.43	0.91	0.60	0.75	0.32	0.58	0.46	0.52	0.12
	DmFc	0.37	0.28	0.33	0.09	0.19	0.07	0.13	0.12	0.28	0.18	0.23	0.11			0.00	0.00
**Ce(NP^{t}Bu(pyrr)2)4**	DmFc v Fc			-0.50				-0.56				-0.53					
	Shift Fc					-0.02	-0.25	-0.14						Diff:		0.23	
	Shift DmFc					-0.18	-0.21	-0.20									

		Before				After				Average				Internal			
		Ep_a	Ep_c	$E_{1/2}$	ΔE	Ep_a	Ep_c	$E_{1/2}$	ΔE	Ep_a	Ep_c	$E_{1/2}$	ΔE	Ep_a	Ep_c	$E_{1/2}$	ΔE
	Fc	0.90	0.47	0.69	0.43	0.85	0.46	0.66	0.39	0.88	0.47	0.67	0.41	1.80	1.50	1.65	0.30
	DmFc	0.19	0.07	0.13	0.12	0.20	0.09	0.15	0.11	0.20	0.08	0.14	0.12			0.00	0.00
**U(NP^{t}Bu(pyrr)2)4**	DmFc v Fc			-0.56				-0.51				-0.53					
	Shift Fc					-0.05	-0.01	-0.03						Diff:		0.98	
	Shift DmFc					0.01	0.02	0.02									

		Bare				Frit				Average			
		Ep_a	Ep_c	$E_{1/2}$	ΔE	Ep_a	Ep_c	$E_{1/2}$	ΔE	Ep_a	Ep_c	$E_{1/2}$	ΔE
	Fc	0.87	0.81	0.84	0.06	0.83	0.47	0.65	0.36	0.85	0.64	0.75	0.21
	DmFc	0.36	-0.12	0.12	0.48	0.31	-0.15	0.08	0.46	0.34	-0.14	0.10	0.47
**Fc**	DmFc v Fc			-0.72				-0.57				-0.65	
	Shift Fc					-0.04	-0.34	-0.19					
	Shift DmFc					-0.05	-0.03	-0.04					

References

(1) Otte, K. S.; Niklas, J. E.; Studvick, C. M.; Boggiano, A. C.; Bacsa, J.; Popov, I. A.; La Pierre, H. S. Divergent Stabilities of Tetravalent Cerium, Uranium, and Neptunium Imidophosphorane Complexes. *Angew. Chem. Int. Ed.* **2023**, e202306580. DOI: https://doi.org/10.1002/anie.202306580.

(2) Niklas, J. E.; Studvick, C. M.; Bacsa, J.; Popov, I. A.; La Pierre, H. S. Ligand Control of Oxidation and Crystallographic Disorder in the Isolation of Hexavalent Uranium Mono-Oxo Complexes. *Inorg. Chem.* **2023**, *62* (5), 2304-2316. DOI: 10.1021/acs.inorgchem.2c04056 From NLM PubMed-not-MEDLINE.

(3) Dutkiewicz, M. S.; Goodwin, C. A. P.; Perfetti, M.; Gaunt, A. J.; Griveau, J.-C.; Colineau, E.; Kovács, A.; Wooles, A. J.; Caciuffo, R.; Walter, O.; et al. A terminal neptunium(V)–mono(oxo) complex. *Nat. Chem.* **2022**, *14* (3), 342-349. DOI: 10.1038/s41557-021-00858-0.

(4) Mikeska, E. R.; Blakemore, J. D. Evidence for Reactivity of Decamethylcobaltocene with Dichloromethane. *Organometallics* **2023**, *42* (13), 1444-1447. DOI: 10.1021/acs.organomet.3c00176.
